# Genomic and evolutionary portraits of disease relapse in acute myeloid leukemia

**DOI:** 10.1038/s41375-021-01153-0

**Published:** 2021-02-12

**Authors:** Franck Rapaport, Yaseswini Neelamraju, Timour Baslan, Duane Hassane, Agata Gruszczynska, Marc Robert de Massy, Noushin Farnoud, Samuel Haddox, Tak Lee, Juan Medina-Martinez, Caroline Sheridan, Alexis Thurmond, Michael Becker, Stefan Bekiranov, Martin Carroll, Heardly Moses Murdock, Peter J. M. Valk, Lars Bullinger, Richard D’Andrea, Scott W. Lowe, Donna Neuberg, Ross L. Levine, Ari Melnick, Francine E. Garrett-Bakelman

**Affiliations:** 1 Molecular Cancer Medicine Service, Human Oncology and Pathogenesis Program, Memorial Sloan Kettering Cancer Center, New York, NY, USA; 2 Center for Clinical and Translational Science, The Rockefeller University, New York, NY, USA; 3 St. Giles Laboratory of Human Genetics of Infectious Diseases, The Rockefeller University, New York, NY, USA; 4 Department of Biochemistry and Molecular Genetics, University of Virginia School of Medicine, Charlottesville, VA, USA; 5 Cancer Biology and Genetics Program, Sloan Kettering Institute, Memorial Sloan Kettering Cancer Center, New York, NY, USA; 6 Division of Hematology/Oncology, Weill Cornell Medicine, New York, NY, USA; 7 Department of Medicine, University of Rochester, Rochester, NY, USA; 8 Division of Hematology and Oncology, University of Pennsylvania Perelman School of Medicine, Philadelphia, PA, USA; 9 Department of Hematology, Erasmus MC Cancer Institute, University Medical Center Rotterdam, Rotterdam, the Netherlands; 10 Department of Internal Medicine III, University Hospital of Ulm, Ulm, Germany; 11 Department of Hematology, Oncology and Tumor Immunology, Charité University Medicine Berlin, Berlin, Germany; 12 Centre for Cancer Biology, University of South Australia and SA Pathology, Adelaide, SA, Australia; 13 Howard Hughes Medical Institute, Chevy Chase, MD, USA; 14 Department of Data Science, Dana Farber Cancer Institute, Boston, MA, USA; 15 Department of Medicine, University of Virginia School of Medicine, Charlottesville, VA, USA; 16 University of Virginia Cancer Center, Charlottesville, VA, USA

## To the Editor:

Relapse in acute myeloid leukemia (AML) patients remains a clinical challenge. The majority of AML patients who receive induction treatment with combination chemotherapy achieve clinicopathologic remission. However, a significant proportion of these patients will relapse and succumb to chemoresistant disease [1]. The biological mechanisms that contribute to relapsed AML are yet to be fully deciphered. Previous studies investigating genetic contributions to AML disease relapse included small numbers of patient samples and/or focused on a small number of AML subtypes. These studies have suggested that disease relapse is associated with founder clone recurrence, subclonal expansion and/or the occurrence of relapse-specific events (reviewed in [2]). To better understand the somatic genomic changes that drive AML relapse, we analyzed specimens (*n* = 120) from a clinically annotated adult relapsed AML patient cohort [3] (Supplementary Table S1, Supplementary Fig. S1) for somatic events. The median age of the patient cohort was 50 years. All patients received standard of care combination chemotherapy, achieved complete remission and experienced disease relapse.

**Fig. 1:**
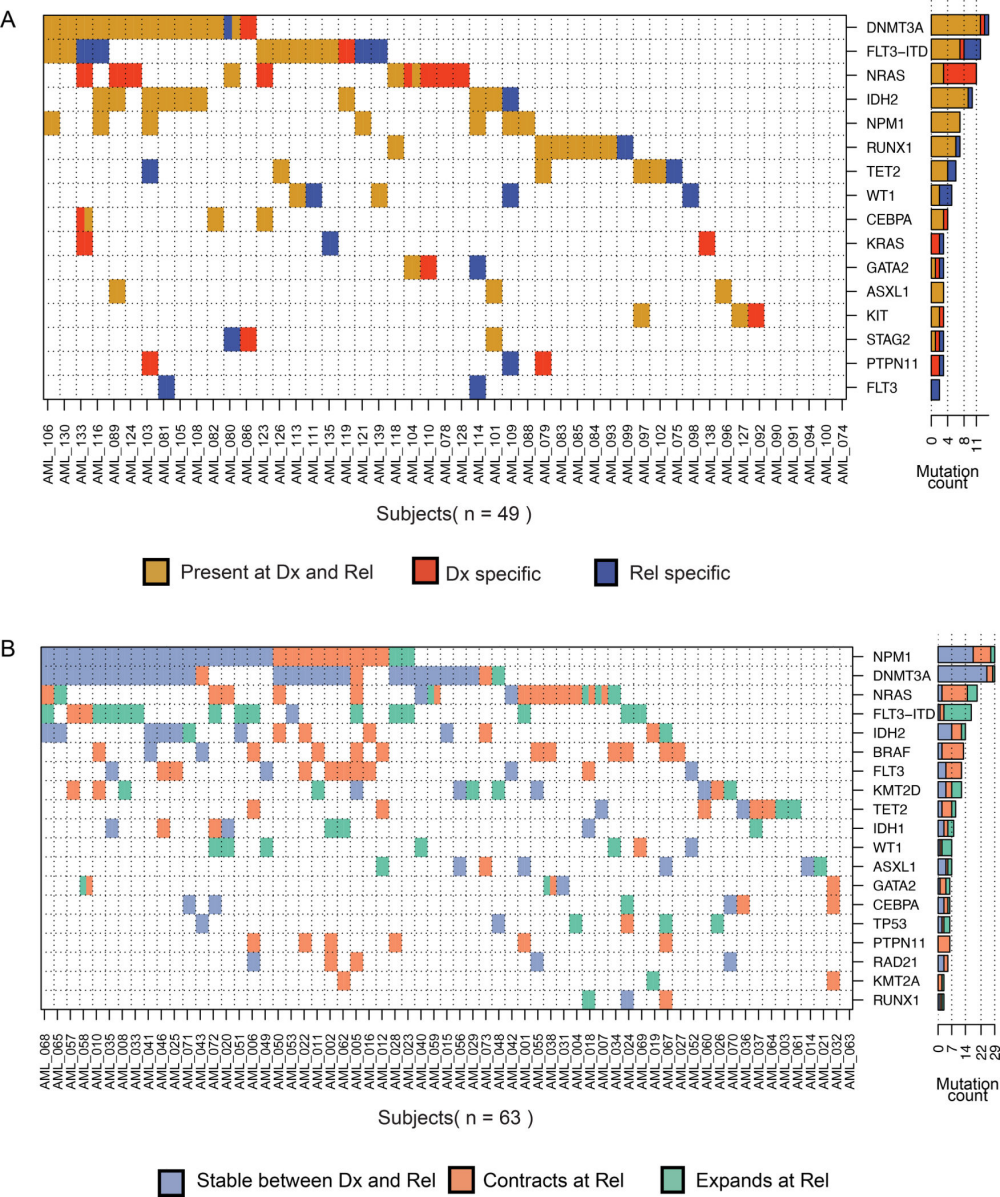
Genomic landscape of relapsed AML. **A** Comutation map for the whole exome sequencing cohort. Each row is a gene and each column a patient. Mutations were summarized by gene with the exception of FLT3-ITD independently plotted. A cell is colored if the corresponding gene is mutated in the corresponding patient. Every gene that is mutated in at least three patients is included. The bar plot shows the number of patients for which we detected a mutation in this gene. Colors: brown = events detected in both diagnosis and relapse, red = events only detected at diagnosis, and blue= events only detected at relapse. **B** Co-mutation map in the targeted panel cohort. Each cell is colored blue if the event is found stable between diagnosis and relapse, orange if it significantly contracts between diagnosis and relapse, and green if it significantly expands between diagnosis and relapse.

We first reanalyzed whole exome sequencing (diagnosis, relapse and matched germlines) of 49 patients [3] in order to capture the complete intragenic mutational burden (Fig. 1A, Supplementary Tables S2 and S3). 21 patients had at least one mutation lost at relapse. Twenty-three patients gained at least one mutation at relapse. A subset of recurrent somatic mutations were validated using orthogonal sequencing (Supplementary Fig. S2; Supplementary Tables S4 and S5). In addition to previously reported commonly mutated genes [4, 5], we identified recurrently mutated genes (at least two patients) that were stable or gained upon disease relapse. Other mutations impacted chromatin remodeling *(ARID1B, BCORL1, CREBBP)* and chromatid cohesion *(ESPL1)* (Supplementary Tables S2 and S3). Previously, mutations in chromatin-related genes at diagnosis were reported to associate with higher rates of relapse [6].

To further understand the patterns of disease progression, we performed copy number alteration (CNA) analyses using sparse whole genome sequencing in paired patient specimens (*n =* 69; Supplementary Fig. S3). Results were compared to clinical cytogenetics data and two specimens were removed from the analysis due to discrepant findings. 44.7% of the 67 patients assessed (*n* = 30) had no detectable CNAs (Supplementary Table S6). In the remaining patients, 34 events were gained and 14 were lost at relapse. A high number of CNAs (three or more unrelated events) was present in 14.9% of the patients (*n* = 10): three with CNAs at both diagnosis and relapse, two with diagnosis-specific events, and five with CNAs gained upon relapse. Four of the five cases presented with “atypical” Complex Karyotype disease and were not associated with *TP53* mutations [7]. The remaining case exhibited a *TP53* R273H mutation that increased in allelic frequency from 0.0864 at diagnosis to 0.281 at relapse with sparse sequencing data revealing associated deletions at 5q and 17p among others (Supplementary Fig. S3; Supplementary Tables S2, S3 and S6). These karyotype changes are in agreement with a previous report revealing changes in disease karyotypes upon disease relapse [8].

To identify genetic variation associated with subclone expansion or contraction during disease progression, we implemented a targeted panel sequencing experiment on 63 matched diagnosis and relapse patient specimens. We focused on 38 genes frequently mutated in AML, previously reported as oncogenic and likely-oncogenic somatic events [6] (Supplementary Tables S2b and S7). Genetic variation was considered significantly higher or lower if the difference in allele fraction at relapse compared to diagnosis was at least 0.05 VAF with a significance of *p* < 0.05 in a Fisher statistical test (Supplementary Table S7). In more than 50% of the patients that had a mutation in *TP53, WT1* or the canonical FLT3-ITD, the mutant subclone expanded at relapse compared to diagnosis (Fig. 1B). By contrast, more than 50% of the subclones with MAPK activating mutations (e.g., *NRAS, PTPN11,* and non-ITD *FLT3)* contracted at relapse (Fig. 1B). Interestingly, in two patients, a sub-clonal *NRAS* mutation at the time of diagnosis was lost yet they gained another subclonal mutation in the same gene at relapse. Mutations in *CEBPA, DNMT3A,* and *NPM1* were more often associated with a clonal fraction that was stable between diagnosis and relapse (Fig. 1B).

**Fig. 2:**
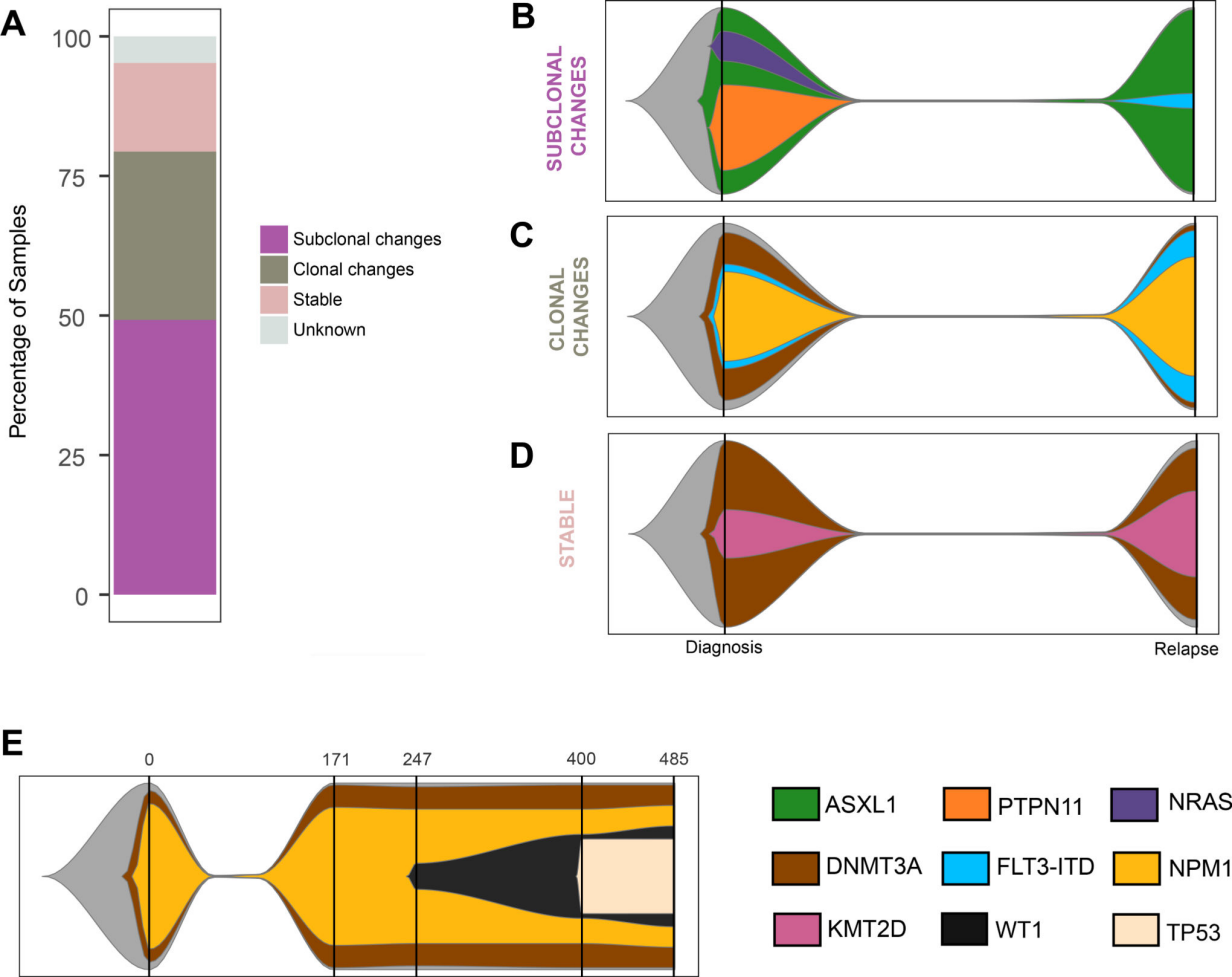
Clonal evolution during disease progression (targeted panel sequencing cohort). **A** Partition of the targeted panel sequencing cohort into each of the evolution patterns. Graphical representations of examples of each evolution pattern identified: subclonal (**B**: AML_001), clonal changes (**C**: AML_023), and stable changes (**D**: AML_029) using a fish plot representation. The clone with the highest VAF at a given time point was considered the parent clone. Subclones were defined based on criteria detailed in the Clonal evolution analysis subsection of “Materials and Methods”. The color key for gene contributions to the pattern is located in the lower right corner of the figure. E Graphical representation of AML_130 tumor evolution pattern. Each vertical bar indicates a tumor sample collection time point, with the time point (in days) along the *x*-axis.

We next determined inferred clonal evolution for each patient of the targeted panel sequencing cohort between diagnosis and relapse. Sixty of the patients could be divided into three groups based on the greatest magnitude of change (Fig. 2A; Supplementary Table S8). Group 1: Subclonal changes: 31 patients exhibited significant change(s) in subclonal composition (Supplementary Fig. S4; representative examples in Fig. 2B and Supplementary Fig. S5). Group 2: Clonal changes: 19 patients had either a conversion of at least one subclonal fraction at diagnosis into a clonal event at relapse or a de novo clonal event at relapse (Supplementary Fig. S6; representative example in Fig. 2C. and Supplementary Fig. S7). Group 3: Stable: ten patients had no significant difference observed (Supplementary Fig. S8; representative example in Fig. 2D). In three cases, we could not reconcile the changes between the diagnosis and relapse samples, suggesting either complex dynamics not explained by the models, or the presence of uncommon events outside of the targeted regions.

For three of the patients included in the study, serial specimens were available for further clonal progression assessment (Supplementary Tables S2c and S9). Results were consistent with stable disease after first relapse (AML_124 and AML_126; Supplementary Fig. S9) and the possibility of further subclonal changes during disease progression (AML_130; Fig. 2E). These data further support the occurrence of the proposed evolution models observed throughout the disease time course.

We previously reported that shifts in DNA methylation heterogeneity could classify patients who progress from diagnosis to relapse [3]. We did not find any significant association between the genomic evolution patterns and DNA methylation heterogeneity groupings (Kruskal–Wallis test, *P =* 0.433). Furthermore, patients’ age, sex, ELN classification [9], treatment type, and time to relapse did not significantly associate with the genomic evolution classifications (Kruskal–Wallis test, *P* > 0.05; Supplementary Table S10).

Our work suggests that clonal dynamics can potentially contribute to therapeutic resistance and disease progression. Our evolution model predictions are similar to those originally reported from mutational or cytogenetics data [2]. However, we cannot exclude the possibility that alternative drivers of clonal composition were not detected in our data, nor that different treatments will associate with different clonal evolution patterns. Interestingly, the lack of association between epigenetic and genetic evolution progression patterns further supports an independent role for each process during disease progression and the potential for parallel approaches cells can take to disease diversification [3].

Our data suggests that subclonal changes could be pathogenic in the etiology of AML relapse. Expansion of clones with *FLT3*-ITD at relapse suggests that this enrichment may contribute to disease progression potentially via STAT5 activation, enhanced cell proliferation and/or differentiation blockade [10]. Likewise, expansion of *WT1* mutations in a subset of patients may contribute to transcriptional dysregulation and impaired hematopoietic differentiation associated with leukemogenesis [11] or to resistance to treatment with DNA damage agents possibly through disrupted TP53 stabilization and transcriptional activity [12]. Finally, our data suggesting the loss of subclones with MAPK activator gene mutations support previous findings consistent with *NRAS* mutations predisposing leukemic cells to cytarabine-induced differentiation [13]. Changes in *FLT3-ITD* and karyotype also represent a potential important clinical consideration for treatment of relapsed disease with targeted [14] or PLK1-directed therapy [15]. Importantly, the fact that actionable driver mutations present at diagnosis can be lost or gained at relapse supports a role for temporal monitoring to inform clinicians about possible personalized targeted therapies to consider to maximize clinical benefits in relapsed AML patients.

## Supplementary Material

1680211_SuppTable1

1680211_SuppTable2

1680211_SuppTable3

1680211_SuppTable4

1680211_SuppMaterial

1680211_SuppTable6

1680211_SuppTable7

1680211_SuppTable8

1680211_SuppTable9

1680211_SuppTable11
