# Supplementary material for: Genomic and evolutionary portraits of disease relapse in acute myeloid leukemia
Source: Leukemia. 2021 Feb 12;35(9):2688–92. doi: 10.1038/s41375-021-01153-0 (PMC8357838; doi:10.1038/s41375-021-01153-0)
Supplement: Supplementary file 1 — Supplementary materials [file 41375_2021_1153_MOESM1_ESM.pdf]

Genomic and evolutionary portraits of disease relapse in Acute Myeloid Leukemia

Supplementary materials

Materials and methods **3**

Patient characteristics and sample processing information 3

Exome capture 4

Targeted resequencing 4

Validation of somatic mutations 5

Copy number aberration data 5

Analysis & genome versioning 6

Clonal evolution analysis 6

Data deposition 7

Assessing association with clinical parameters 7

Supplementary Figures **8**

Supplementary Figure 1 8

Supplementary Figure 2 9

Supplementary Figure 3 10

Supplementary Figure 4 11

Supplementary Figure 5 17

Supplementary Figure 6 18

Supplementary Figure 7 22

Supplementary Figure 8 23

Supplementary Figure 9 25

Supplementary Tables **26**

Supplementary Table 1: Summary of study subject clinical data. 26

Supplementary Table 2: Somatic mutations determined in study subjects. 26

Supplementary Table 3: Mutations identified in the whole exome sequencing cohort and associated changes during AML disease progression. 26

Supplementary Table 4: Somatic mutations validated in the whole exome sequencing cohort. 26

Supplementary Table 5: IDH1 and IDH2 mutation validations using a sequenom assay. 27

Supplementary Table 6: Copy number aberration analysis results. 28

Supplementary Table 7: Mutations identified in the targeted panel sequencing cohort. 28

Supplementary Table 8: Inferred clonal evolution groups. 28

Supplementary Table 9: Multi-relapse patient specimen information and results. 28

Supplementary Table 10: Kruskal Wallis tests results assessing for association between genomic evolution categories, DNA methylation epiallele categories, and clinical parameters. 29

Supplementary Table 11: Sequencing statistics of the sparse whole genome sequencing assay performed in the study. 30

Supplementary Materials' bibliography **31**

Materials and methods

Patient characteristics and sample processing information

120 clinically annotated, paired Acute Myeloid Leukemia (AML) patient samples (diagnosis and relapse: 47 females and 73 males) from adult patients were utilized for the study. These adult patients (older than 18), which excluded acute promyelocytic leukemia patients, were seen at medical centers in Australia, Germany, Netherlands, and the United States. Patients with acute promyelocytic leukemia were excluded. All patients were treated according to the protocols of corresponding institutes and hospitals. Donors (AML patients) signed informed consent according to the declaration of Helsinki for collection and use of sample materials in research protocols at the following clinical centers: Erasmus Medical Center (protocol number MEC-2015-155), Royal Adelaide Hospital and SA Pathology (Adelaide, South Australia; 1998-onwards), University of Pennsylvania (protocol number 703185), University of Rochester Medical Center (protocol number URCC ULEU07047), and the University Hospital of Ulm. Study protocols were approved by the Institutional Review Boards of corresponding institutes and hospitals (protocols above-noted), and at Weill Cornell Medicine (WCM; protocol number 0805009783) and the University of Virginia (IRB protocol number IRB-HSR 19796). For AML patient cryopreserved specimens from the Royal Adelaide Hospital and SA Pathology (Adelaide, South Australia) collected and stored prior to 1998, the requirement for informed consent was waived by the Royal Adelaide Hospital Human Research Ethics Committee (RAH Protocol #110304b). The use of the samples obtained from RAH and SA Pathology in this specific research study was approved by the RAH HREC on September 10, 2010.
The clinical and molecular characteristics of these patients utilized in this study are included in **Supplementary Table 1**. Cytogenetic analysis at relapse was not part of routine diagnostics at the time of relapse treatment at the clinical centers and was therefore not available. Risk classification was performed per the European Leukemia Network criteria (1). The subjects included in the study are a subset of patients included in Li, Garrett-Bakelman, et al. (2) and processed as described. All patients were treated with combination chemotherapy (cytarabine arabinoside and an anthracycline) during induction phase followed by consolidation chemotherapy treatment with or without a stem cell transplantation in first remission per clinical center standards.
Samples from serial time points were available for study subjects AML_124, AML_126 and AML_130. AML_124 was a 65 year old male. He was treated with daunorubicin and cytarabine induction chemotherapy followed by four treatment cycles of high dose cytarabine for consolidation. Samples were available from the diagnostic time point (AML_124_1), and at the following time points: first relapse (AML_124_2) and refractory relapse following treatment with high dose cytarabine and CEP-701 (AML_124_3). AML_126 was a 60 year old male. He was treated with daunorubicin and cytarabine induction chemotherapy followed by three treatment cycles of high dose cytarabine for consolidation. Samples were available at the following time points: diagnosis (AML_126_1), first relapse (AML_126_2), refractory relapse following one cycle of high dose cytarabine (AML_126_3), and following one cycle of mitoxantrone, etoposide and intermediate dose cytarabine (AML_126_4). All viably frozen samples were thawed and subjected to depletion of lymphocyte (CD3+ and CD19+) cells using magnetic beads (Miltenyi Biotec). DNA was isolated using the blood puregene kit (Qiagen), quality control was performed using standard agarose gel

visualization and DNA was quantified using ThermoScientific's Qubit 2.0 Fluorometer and Qubit-™dsDNA HS Assay Kit (ThermoScientific, Cat. No. Q32854). AML_130 clinical information and processing was described in Li/Garrett-Bakelman, et al. (2) .

Exome capture

Data for exome capture analysis was obtained from dbGap accession number phs001027.v2.p1(2). Data were aligned using the Burrows-Wheeler Aligner (3) to the human genome (UCSC build hg19) and then pre-processed using GATK per recommended best practices. We performed somatic SNV calling by comparing diagnosis and relapse samples to their matched germline specimens with Mutect (4) , Varscan (5), and somaticSniper (6). Indels were determined using Pindel (7) and GATK SomaticIndelDetector (8). Mutations were annotated with Snpeff (9). Mutations considered for analysis were identified by two callers as previously described (10) or called by a single caller and have already been reported previously (either in (11) or in COSMIC). Analysis using these tools was performed using an internally-developed pipeline (MSKCC Bioinformatics Core: https://www.mskcc.org/research/ski/core-facilities/bioinformatics). Results are summarized in **Supplementary Table 2** and included in **Supplementary Table 3**.

Targeted resequencing

Targeted resequencing was performed using established protocols. Data from targeted resequencing (111 genes) of 51 diagnostic specimens was obtained ((11); EGA accession number: EGAS00001000275). 12 additional diagnostic and the matching 63 relapsed samples were profiled using an IDT custom capture panel (catalog number 1016302) per manufacturer's recommendations. Briefly, 200ng of DNA was sheared using a Covaris LE220 sonicator (adaptive focused acoustics). DNA fragments were end-repaired, adenylated, ligated to Illumina sequencing adapters, and amplified by PCR (using 10 cycles). Targeted capture was subsequently performed using 1µg of the DNA library and a custom AML capture probe set. Captured libraries were then enriched by PCR (using 10 cycles). Final libraries were evaluated using fluorescent-based assays including PicoGreen (Life Technologies) or Qubit Fluorometer (invitrogen) and Fragment Analyzer (Advanced Analytics) or BioAnalyzer (Agilent 2100), and were sequenced on an Illumina HiSeq2500 sequencer per manufacturer's recommendations using 2 x 125bp cycles. The reads were aligned to the human genome (UCSC build hg19) using the Burrows-Wheeler Aligner (3) with maximal exact matches. We used the Cancer Genome Project pipeline (https://github.com/cancerit) and compared the tumor samples to a standard cancer-free germline following the pipeline recommendations. Snpeff (9) was used to annotate variants with their functional consequences. We filtered out variants that were present in any of the population groups from the ExAC database (12) with a minor allele frequency of more than 1%. We only considered variants that were either present in at least two samples or classified as oncogenic or likely oncogenic according to the criteria outlined in (11). Results are summarized in **Supplementary Table 2** and included in **Supplementary Tables 7 and 9**.

The 38 recurrently mutated genes that were used for inferred clonal analysis were: *ASXL1* , *BCOR* , *BRAF* , *CBL* , *CEBPA* , *CTNNA1* , *DNMT3A* , *EP300* , *ETV6* , *FLT3* , *IDH1* , *IDH2* , *JAK2* , *KDM6A* , *KIT* , *KRAS* , *MLL* , *MLL2* , *MPL* , *NRAS* , *PHF6* , *PRPF40B*, *PTEN*, *RAD21* , *RUNX1* ,

SF1 , *SF3A1* , *SF3B1* , *SH2B3* , *SOCS1, SRSF2* , *STAG2* , *TET1* , *TET2* , *TP53* , *U2AF1* , *U2AF2* , and *ZRSR2*.

Validation of somatic mutations

Validation of somatic mutations was performed using a custom targeted amplicon panel and sequenom assay. The custom targeted amplicon panel was performed and data was analyzed as previously described (13): Briefly, 1. Library generation and amplification were performed using a low error rate Hi-Fi DNA polymerase according to the Kapa HyperPrep protocol (Kapa Biosystems). 2. Targeted sequencing using a panel of 22 recurrently mutated genes in hematological malignancies was performed using a custom capture probe set targeting all exons (Integrated DNA Technologies; genes included: *ABL1, ASXL1, BCOR, BCORL1, CALR, CBFB, CEBPA, DNMT3A, FLT3, IDH1, IDH2, JAK2, KIT, KMT2A, KRAS, NPM1, NRAS, RARA, RUNX1, TET2, TP53, WT1*) per manufacturer's recommendations. 3. Following targeted enrichment performed per Nimblegen protocols, libraries were sequenced on an Illumina HiSeq 4000 using dual-indexed sample adapters (Integrated DNA Technologies) to a median coverage of 2000x. 4. Reads were trimmed of contaminating adapter sequences and low- 31 quality bases using Trimmomatic v0.32 (trimmed when median Illumina base quality score < 20 over 6 32 nt sliding window). Overlapping paired end reads were merged into a single long consensus read using AdapterRemoval v241 when at least 12 bp overlap was present. 5. The remaining high-quality reads were aligned to the 1000 Genomes Phase 2 human reference genome and decoy contigs (hs37d5) using BWA MEM. Duplicate marking was performed using SamBlaster v0.1.21 and MarkDupsByStartEnd v0.2.1. 6. Single nucleotide variants (SNVs) and insertions/deletions (indels) were detected using VarDict-Java v1.4.6 in single sample mode with indel realignment. 7. Annotation of variants and their functional impact was performed using Variant Effect Predictor (VEP) v8547 and snpEff v4.1g. 8. To identify somatic variants, filtration based on population allele frequency data was applied so as to enrich somatic variants that are not likely inherited. To this end, variants were classified as probable somatic if exhibiting a dbSNP v142 or ExAC adjusted population allele frequency <= 0.25% or a median variant allele fraction (VAF) of 2.5%. Variants that occured at a VAF of 5% were compared to the exome capture results. The Sequenom assay was performed as previously described (14) to validate *IDH1* and *IDH2* mutations which occurred above a VAF of 0.05% in a subset of specimens. Results are included in **Supplementary Tables 4 and 5**.

Copy number aberration data

Copy number aberration data (CNA) was generated and sequenced (75 bp single-end reads) as previously described (15, 16) (**Supplementary Table 11**). The resulting data was first analyzed by FastQC (17) to determine if it was of sufficient quality for further analysis. Reads were trimmed of adapters using Cutadapt (18) and mapped to the human reference genome hg19 with Burrows Wheeler Aligner (3) using the 'mem' command (BWA-MEM). Aligned reads were converted from SAM to BAM format, sorted, and indexed with Samtools (19). Duplicate reads were removed with MarkDuplicates from Picard Tools (20). 25853 genomic bins, each containing 100kb of nonoverlapping mappable sequence, were defined based on the hg19 mappability track (wgEncodeCrg-

MapabilityAlign75mer.bigWig, UCSC Genome Browser). Next, reads were excluded from unmappable regions and counted in bins with Bedtools (21). The bin counts were then normalized to copy number estimates as follows: bins were sorted into 100 evenly sized groups according to GC content; the median read count was calculated for each GC group; each bin count was divided by the GC group median and then multiplied by 2. Next, to detect outliers, preliminary segments were defined with Circular Binary Segmentation (CBS)(22). The R package DNAcopy (23), which implements CBS, was used with alpha=0.001, min.width = 5, and undo.SD=1. Outliers were removed using Tukey's Outlier Method on the median of differences between bin value and preliminary segment value. Final segments were defined through a second segmentation by DNAcopy following outlier bin removal. To define thresholds for gains and losses, the distribution of segment copy numbers was inspected. For the paired AML cohort, a distribution of segments with length below the sum of median length plus two median absolute deviations (median + 2*MAD) was used. For the AML patients older than 60, a distribution of segments with bin length below the median length was used. Mixed Gaussian models were fitted to these distributions using R package mixtools (24). Gaussians centered near 2 were chosen to represent the diploid copy number state and were used to determine CNV thresholds. A two- tailed cumulative probability of 0.05 resulted in cutoffs equal to 1.73 (loss) and 2.36 (gain). Tools utilized for the analysis: R version 3.5.0: DNAcopy, mixtools, ggplot2, stringr, dplyr, fastqc version 0.11.5, cutadapt version 1.16, bwa version 0.7.17, samtools version 1.7, bedtools version 2.26.0, picard tools version 2.18.5, and java version 1.8.0. Results were subjected to manual review for confirmation. We assessed the copy number calls at the 5k, 20k and 50k bin resolutions and only considered an event true if it was found in at least one resolution. CNVs identified in both analysis pipelines are reported in **Supplementary Table 6**.

Analysis & genome versioning

R version 3.4.4 was used for all analyses unless otherwise specified. Reference genome used for annotation in all analyses was hg19.

Clonal evolution analysis

For each sample of the Targeted resequencing cohort, if no oncogenic or likely oncogenic mutation had a VAF > 0.4, we corrected for tumor content by considering that the highest likely oncogenic or oncogenic mutation was clonal (VAF = 0.5) and proportionally adjusting depth of sequencing and VAF for every mutation of that sample. We considered that a mutation was associated with a clone that contracted or expanded if the VAF of the mutation at relapse was lower or higher, respectively, than the VAF of the mutation at diagnosis by at least 0.05. The difference between the two VAFs was also required to associate with a p<0.05 according to a Fisher's exact test. We represented the changes in VAF with line plots where we represent the 95% confidence interval for each VAF using the function "binconf" from the "Hmisc" package (version 4.4-1; CRAN https://cran.r-project.org/web/packages/Hmisc/Hmisc.pdf).

We considered that two mutations in the same patient belonged to different subclones if their VAF were different at diagnosis or relapse according to a Fisher's exact test (p<0.05) or they had

different directions of evolution (for example, one was contracting and the other one was expanding between diagnosis and relapse).
Changes in the clonal architecture in our dataset are represented using fishplots generated using the “fishplot” R package (version 0.4; github https://github.com/chrisamiller/fishplot). VAFs (**Supplemental tables 7 and 9**) were used to plot the clonal changes. VAFs less than 1 percent in either diagnosis or relapse were considered 0 in the fraction table. All the VAFs are multiplied by 2 to account for heterozygosity and summed up to 100% at each time point . The clone with highest VAF at a given time point was considered the parent clon**e**.

Data deposition

Raw data files were used from previously deposited data into EGA (EGAS00001000275) and dbGap (phs001027.v2.p1). Newly generated sparse whole genome sequencing and targeted resequencing data from all analyses have been deposited into dbGap (phs001027.v3.p1).

Assessing association with clinical parameters

The association of evolutionary models with age , gender , epiallele clusters , treatment type (combination chemotherapy with or without stem cell transplantation), and time to relapse was estimated using the Kruskal Wallis test in R using the “kruskal.test” function.

Supplementary Figures

Supplementary Figure 1

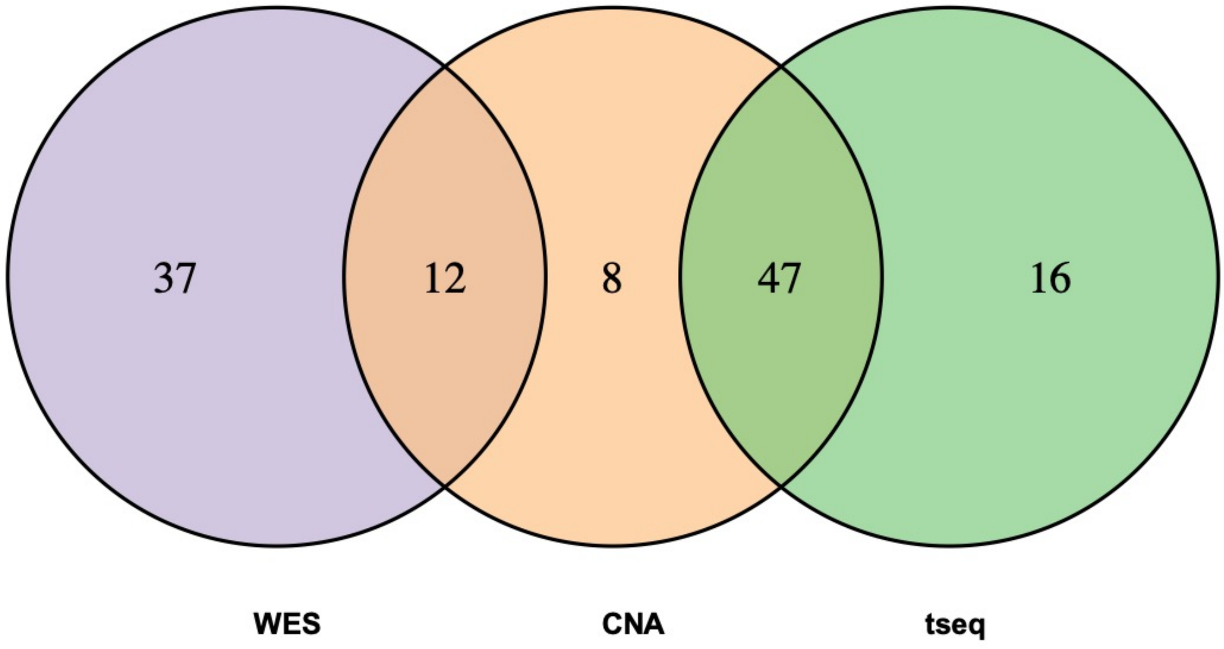

Supplementary Figure 1: Venn diagram of specimens included in the study. Shown is a venn diagram of paired patient specimens included in the study (WES = whole exome sequencing; CNA = copy number alterations analysis, and tseq = targeted panel sequencing).

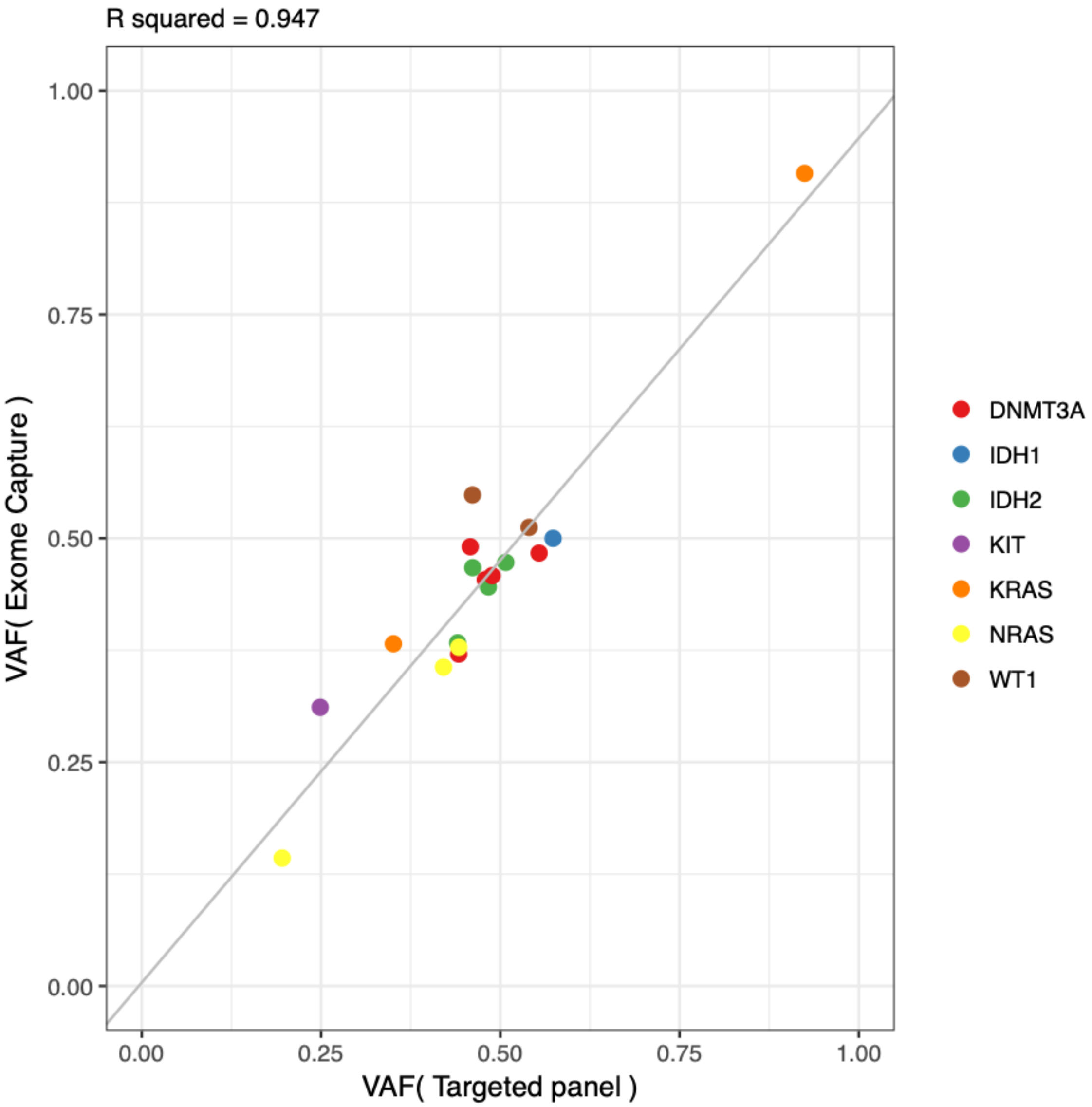

Supplementary Figure 2

Supplementary Figure 2: Validation of somatic mutations identified. Targeted resequencing was performed on a subset of specimens also subjected to exome capture. Common variants identified through these approaches are plotted in a correlation plot of the VAFs determined (R^2 = 0.947).

Supplementary Figure 3

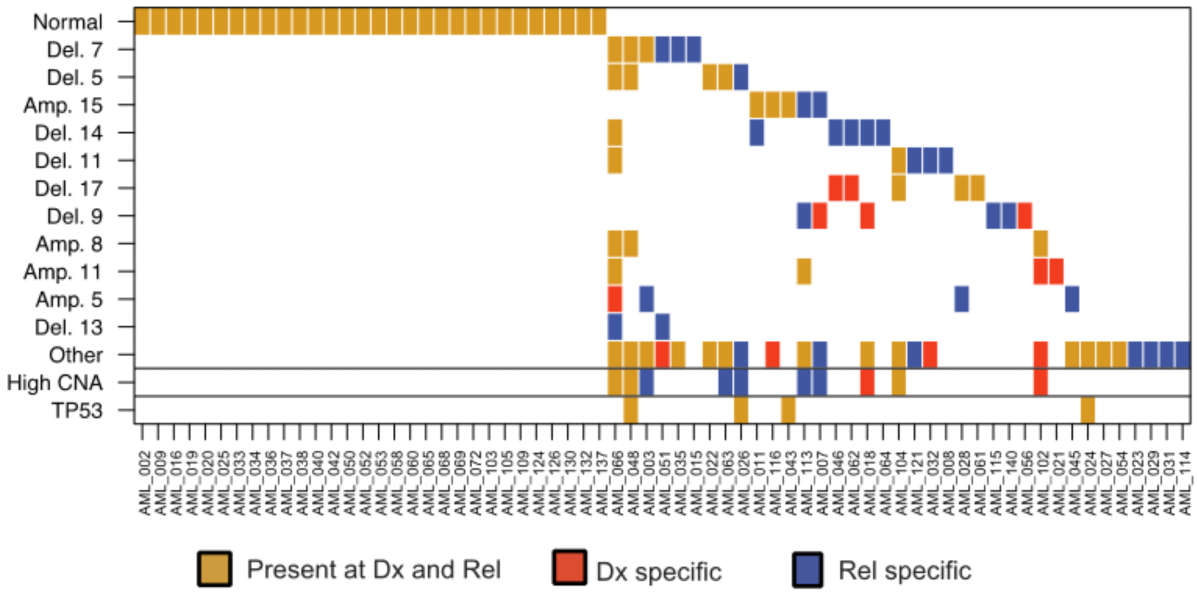

Supplementary Figure 3: Copy Number Aberrations during disease progression. Shown is a co-occurrence plot of copy number aberrations. Each row is a copy number event and each column a patient. The cell is colored if an event was detected in any given patient. Brown, red and blue corresponding to events detected in both diagnosis and relapse, only detected at diagnosis and only detected at relapse respectively. We qualify a specimen as high CNA if we detected three or more distinct copy number events in this patient. The last line indicates the TP53 mutational status of every patient.

Supplementary Figure 4

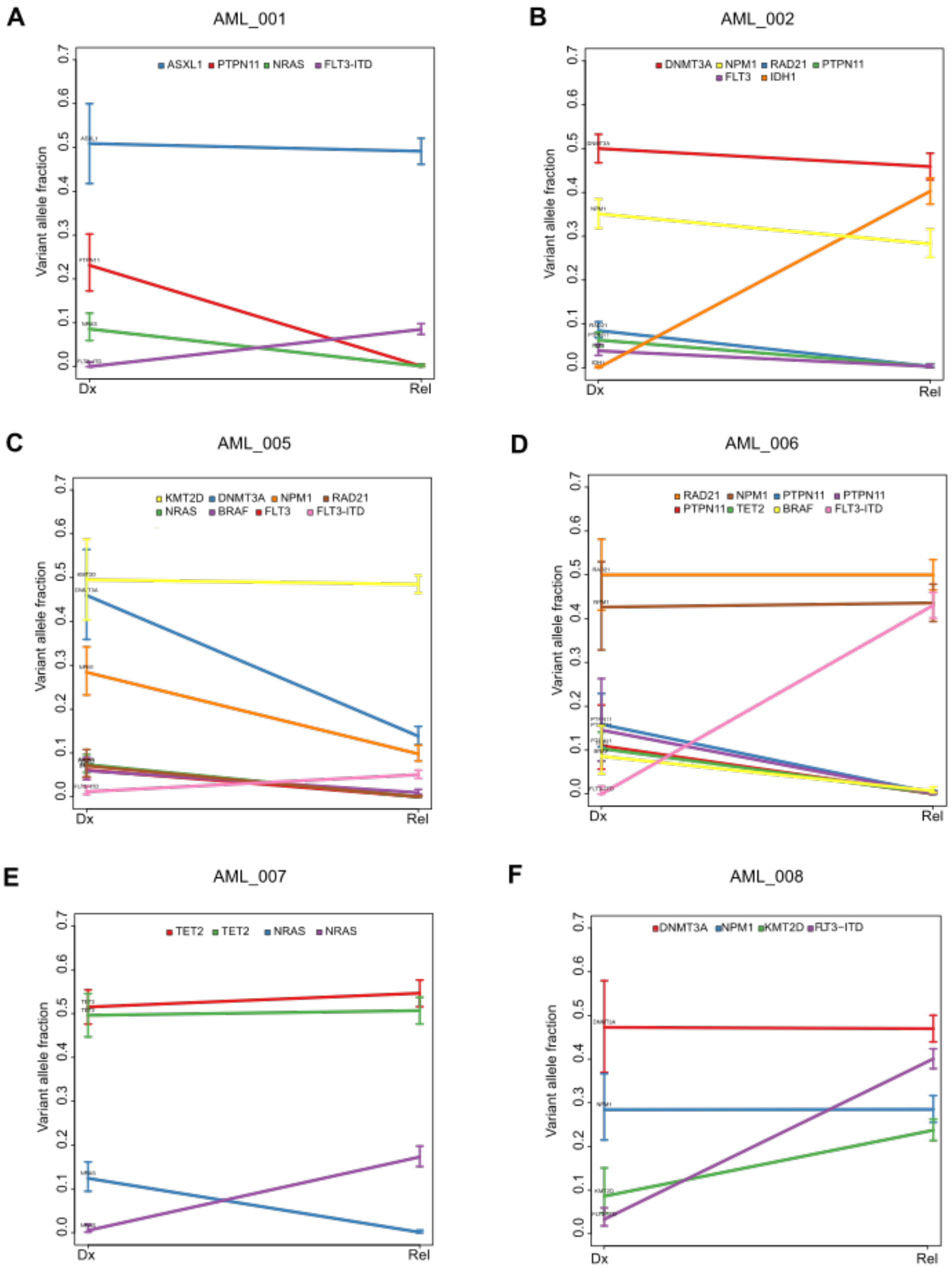

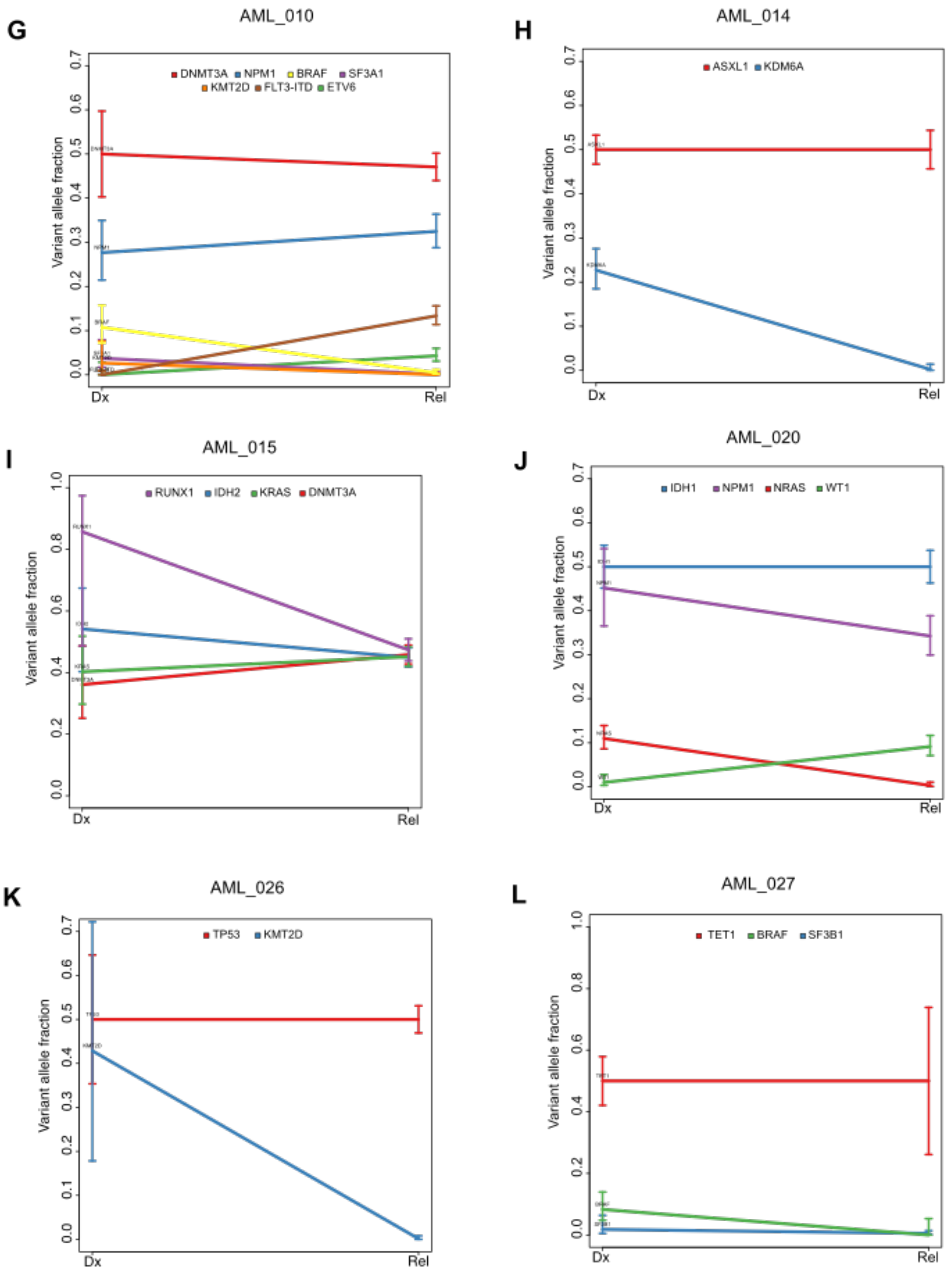
G
AML_010
DNMT3A NPM1 BRAF SF3A1
KMT2D FLT3-ITD ETV6
Variant allele fraction
Dx
Rel
H
AML_014
ASXL1 KDM6A
Variant allele fraction
Dx
Rel
I
AML_015
RUNX1 IDH2 KRAS DNMT3A
Variant allele fraction
Dx
Rel
J
AML_020
IDH1 NPM1 NRAS WT1
Variant allele fraction
Dx
Rel
K
AML_026
TP53 KMT2D
Variant allele fraction
Dx
Rel
L
AML_027
TET1 BRAF SF3B1
Variant allele fraction
Dx
Rel

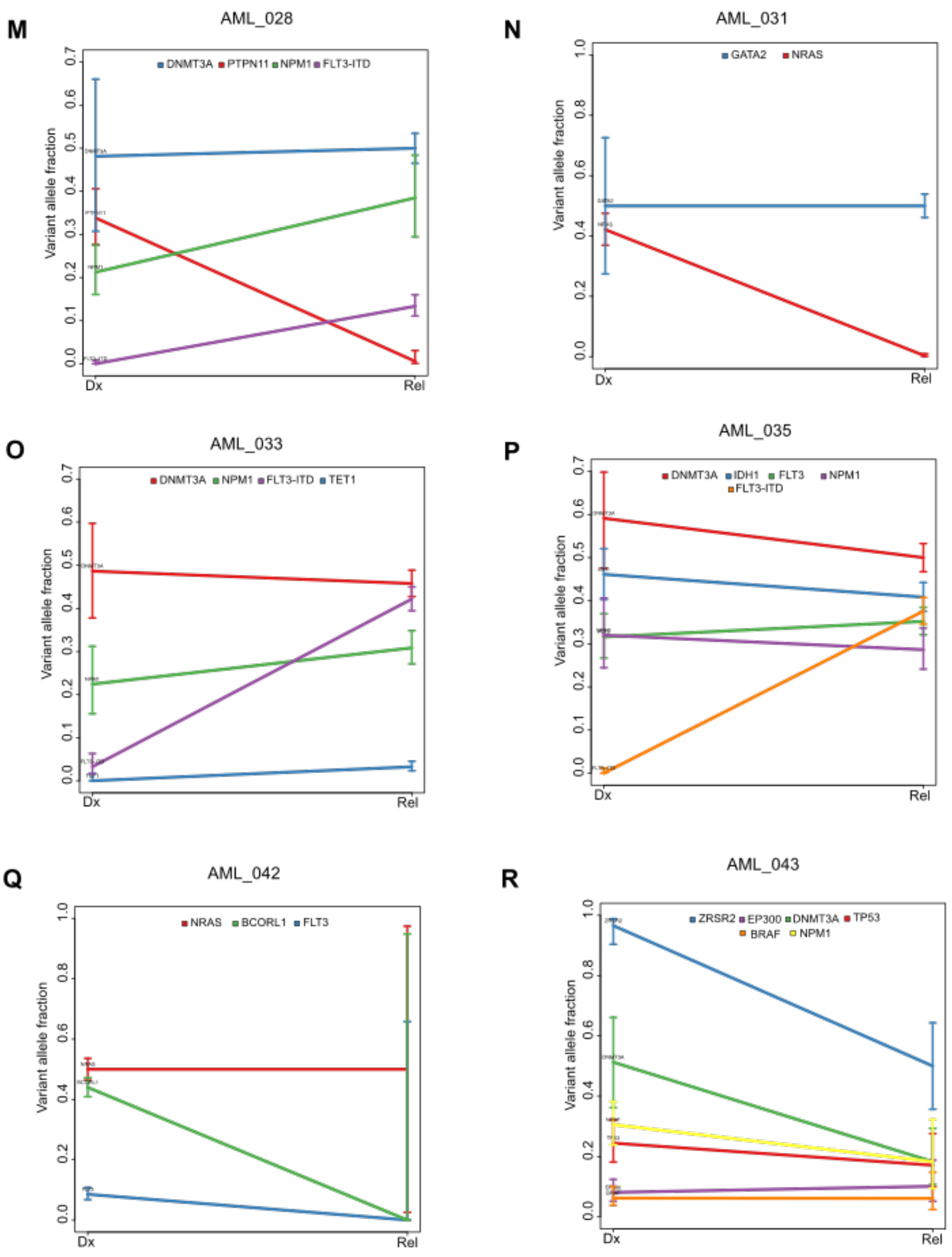
M
AML_028
DNMT3A PTPN11 NPM1 FLT3-ITD
Variant allele fraction
Dx
Rel
N
AML_031
GATA2 NRAS
Variant allele fraction
Dx
Rel
O
AML_033
DNMT3A NPM1 FLT3-ITD TET1
Variant allele fraction
Dx
Rel
P
AML_035
DNMT3A IDH1 FLT3 NPM1
FLT3-ITD
Variant allele fraction
Dx
Rel
Q
AML_042
NRAS BCORL1 FLT3
Variant allele fraction
Dx
Rel
R
AML_043
ZRSR2 EP300 DNMT3A TP53
BRAF NPM1
Variant allele fraction
Dx
Rel

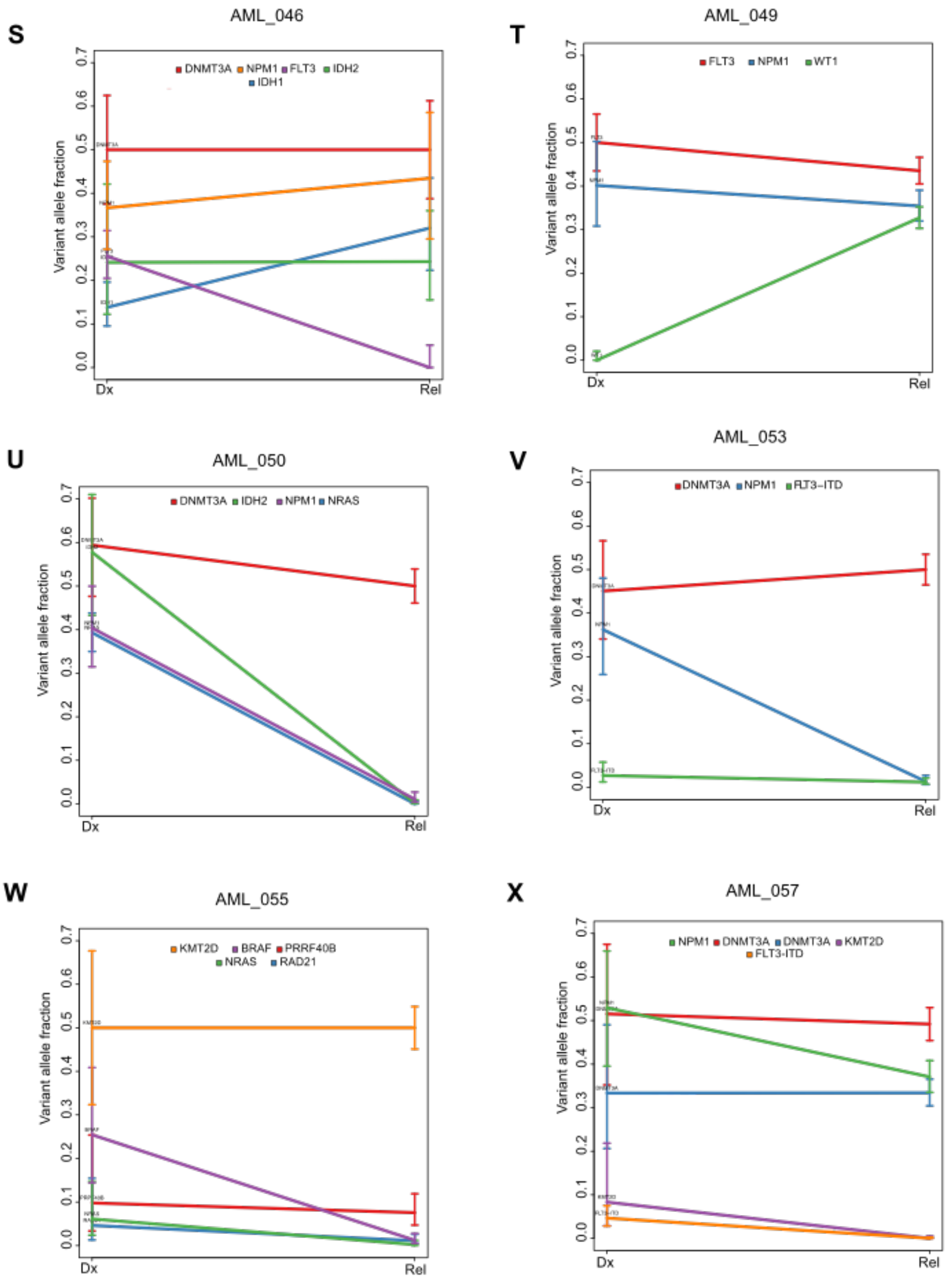
S
AML_046
DNMT3A NPM1 FLT3 IDH2
IDH1
Variant allele fraction
Dx
Rel
T
AML_049
FLT3 NPM1 WT1
Variant allele fraction
Dx
Rel
U
AML_050
DNMT3A IDH2 NPM1 NRAS
Variant allele fraction
Dx
Rel
V
AML_053
DNMT3A NPM1 FLT3−ITD
Variant allele fraction
Dx
Rel
W
AML_055
KMT2D BRAF PRRF40B
NRAS RAD21
Variant allele fraction
Dx
Rel
X
AML_057
NPM1 DNMT3A DNMT3A KMT2D
FLT3-ITD
Variant allele fraction
Dx
Rel

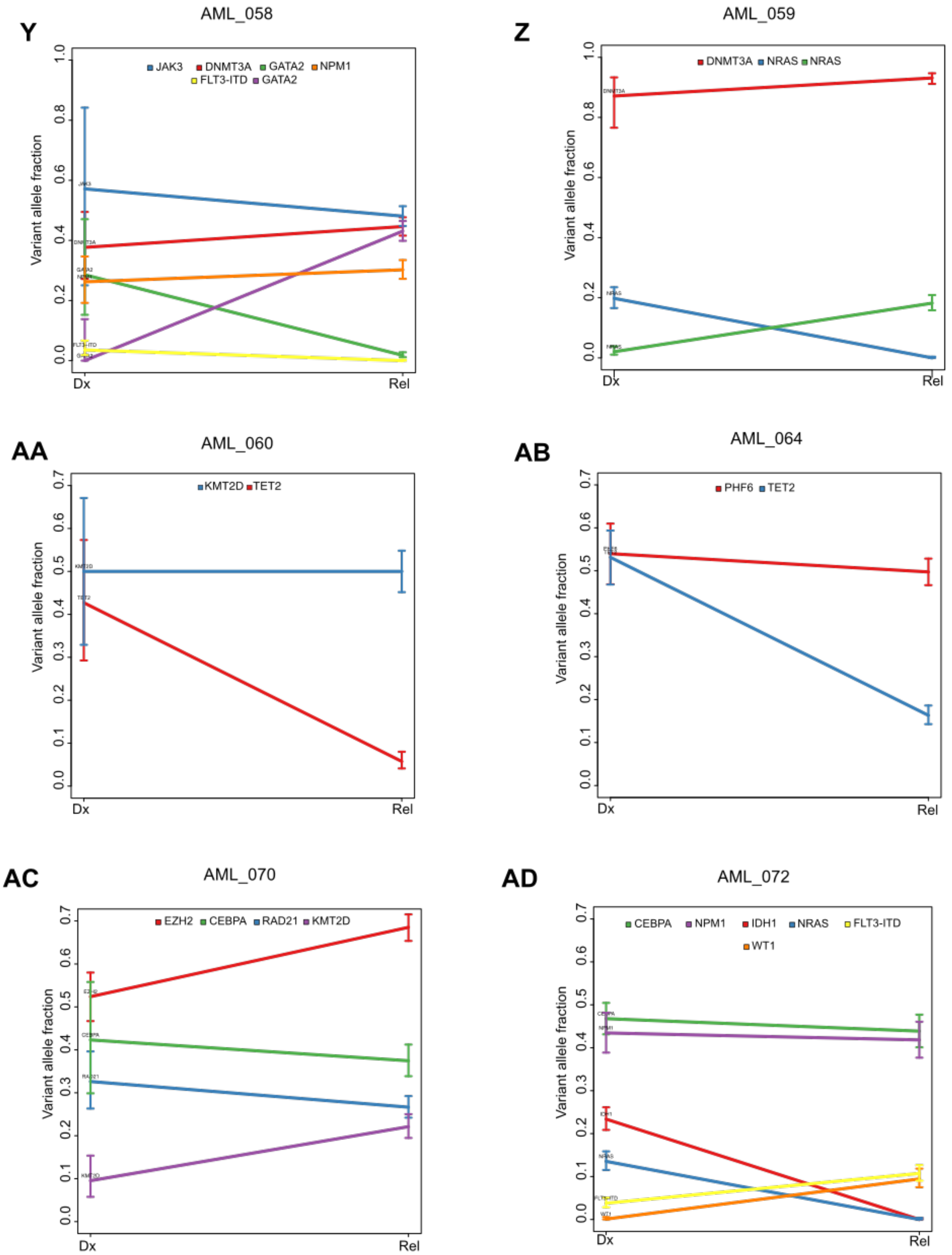
Y
AML_058
JAK3
DNMT3A
GATA2
NPM1
FLT3-ITD
GATA2
Variant allele fraction
Dx
Rel
Z
AML_059
DNMT3A
NRAS
NRAS
Variant allele fraction
Dx
Rel
AA
AML_060
KMT2D
TET2
Variant allele fraction
Dx
Rel
AB
AML_064
PHF6
TET2
Variant allele fraction
Dx
Rel
AC
AML_070
EZH2
CEBPA
RAD21
KMT2D
Variant allele fraction
Dx
Rel
AD
AML_072
CEBPA
NPM1
IDH1
NRAS
FLT3-ITD
WT1
Variant allele fraction
Dx
Rel

AE

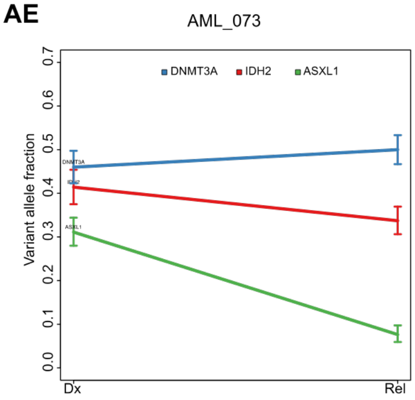

Supplementary Figure 4: Line plots of mutational composition for study subjects with subclonal evolution patterns. VAFs at diagnosis and relapse are plotted for each mutation. Bars denote 95% confidence intervals for the VAF, accounting for the sequencing depth at the associated genomic positions, assuming a binomial distribution of the reads.

Supplementary Figure 5

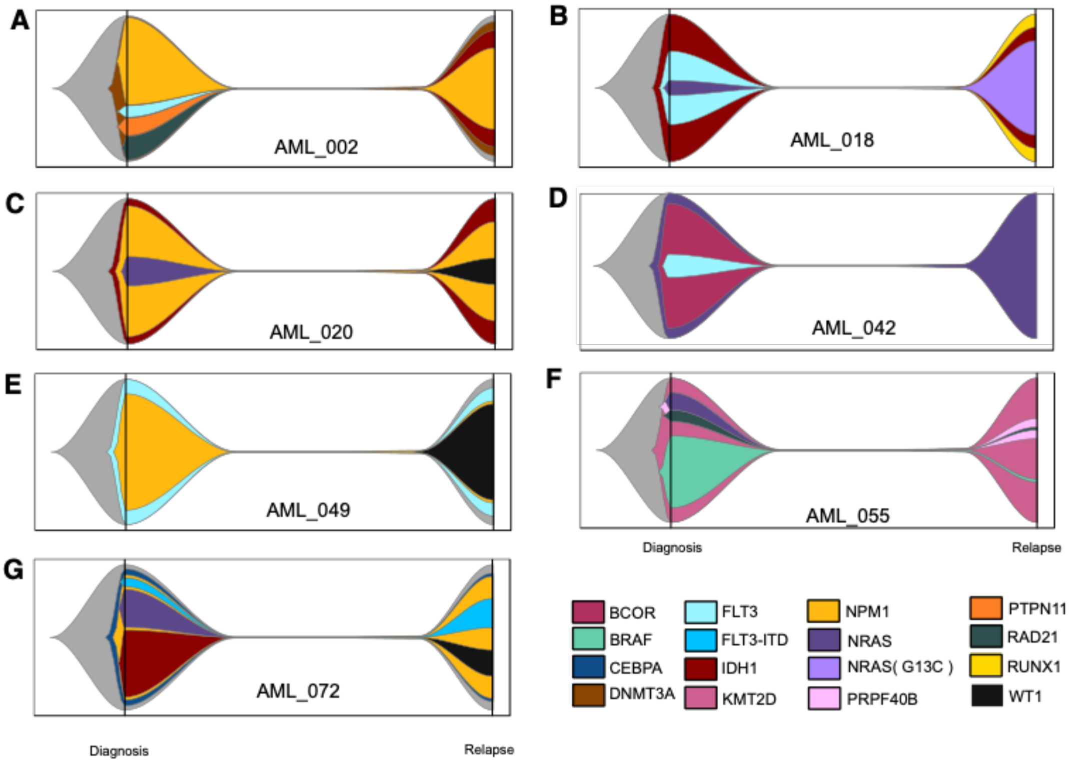

Supplementary Figure 5: Graphical representations of subclonal evolution patterns. Fish plots of representative patients which undergo subclonal evolution between diagnosis and relapse are shown. The color key for gene contributions to the pattern is located in the lower right corner of the figure. Each vertical line indicates a tumor sample at diagnosis or relapse.

Supplementary Figure 6

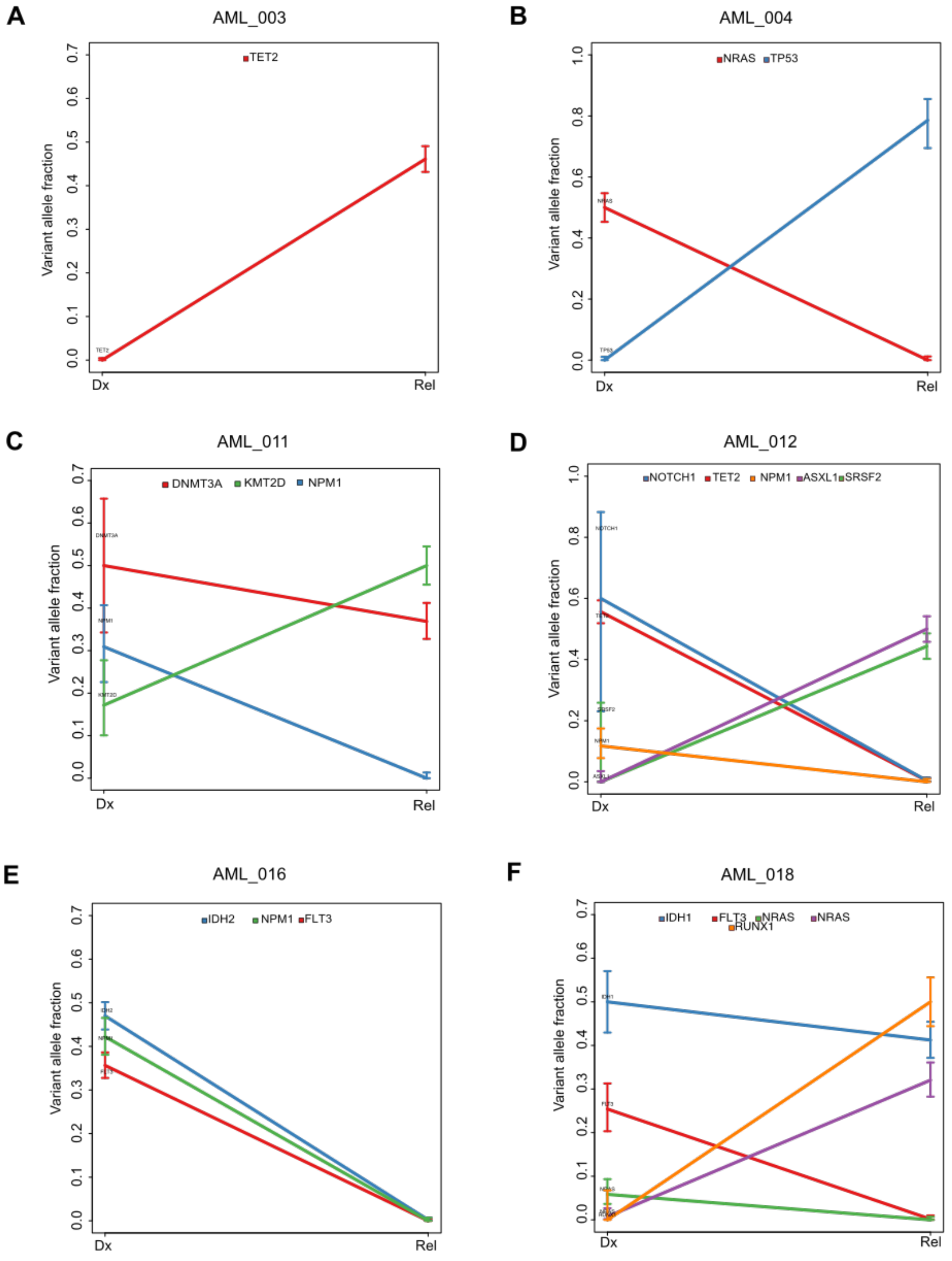

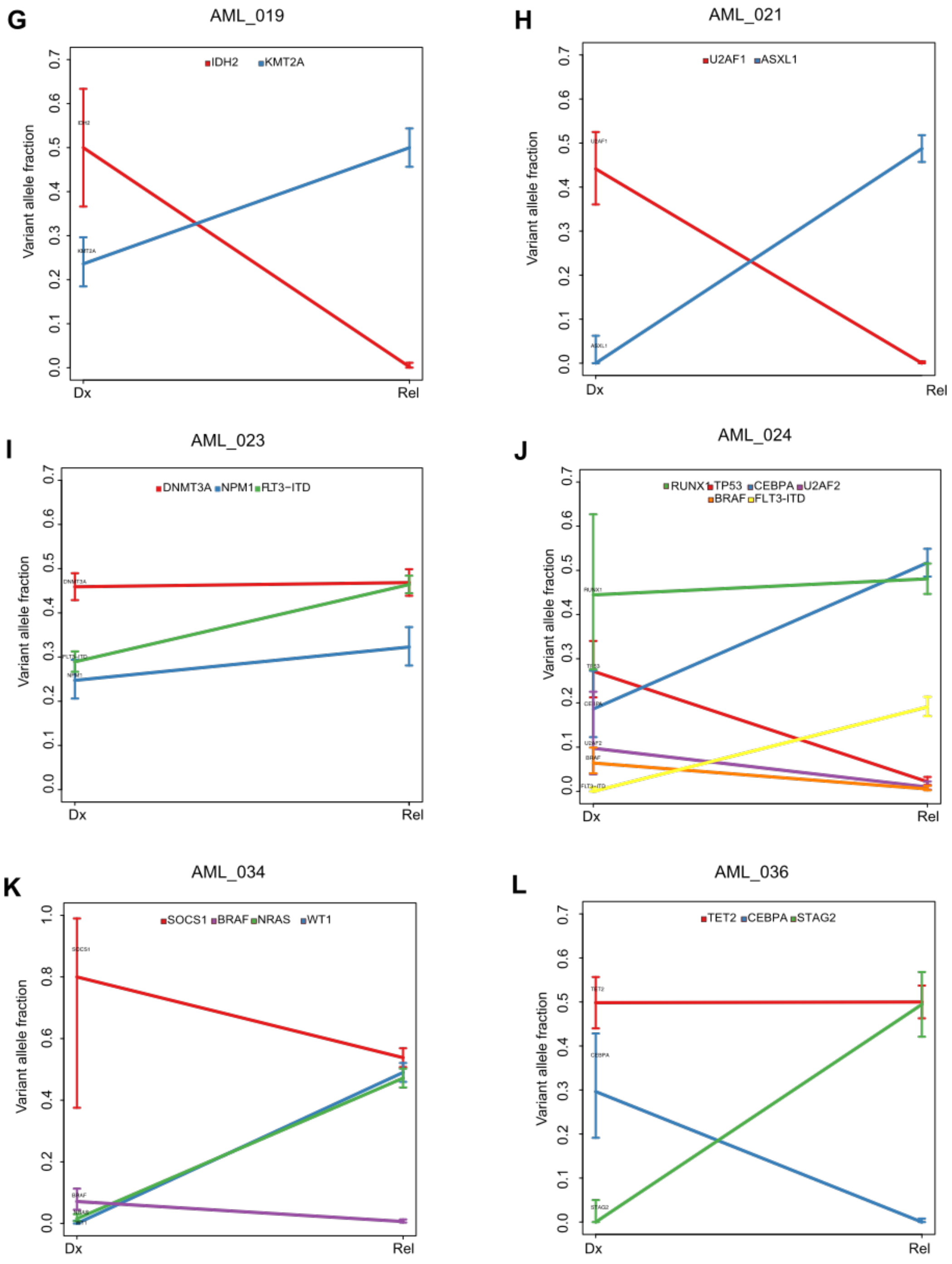
G
AML_019
IDH2
KMT2A
Variant allele fraction
0.0
0.1
0.2
0.3
0.4
0.5
0.6
0.7
Dx
Rel
H
AML_021
U2AF1
ASXL1
Variant allele fraction
Dx
Rel
I
AML_023
DNMT3A
NPM1
FLT3−ITD
Variant allele fraction
Dx
Rel
J
AML_024
RUNX1
TP53
CEBPA
U2AF2
BRAF
FLT3-ITD
Variant allele fraction
Dx
Rel
K
AML_034
SOCS1
BRAF
NRAS
WT1
Variant allele fraction
0.0
0.2
0.4
0.6
0.8
1.0
Dx
Rel
L
AML_036
TET2
CEBPA
STAG2
Variant allele fraction
Dx
Rel

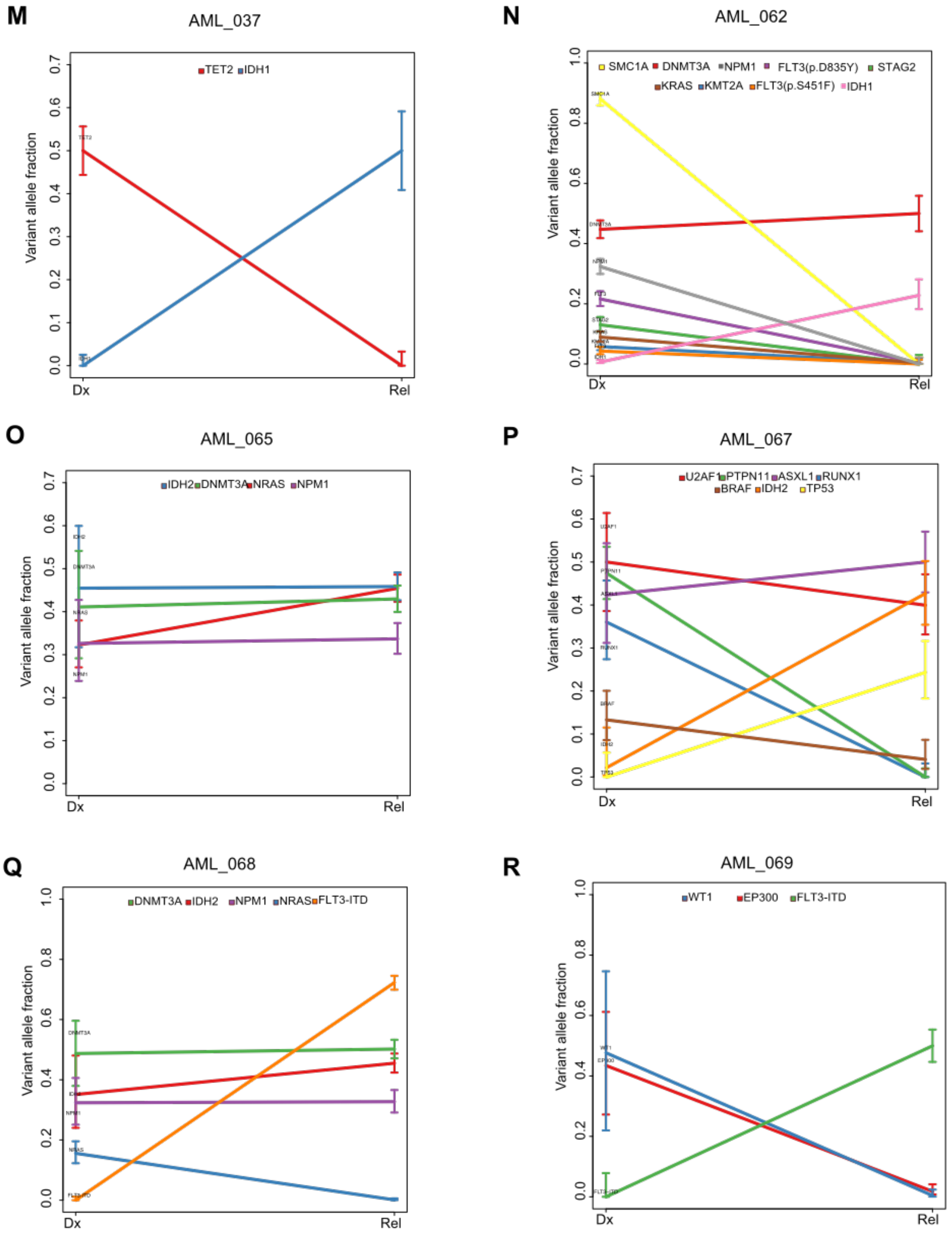
M
AML_037
TET2 IDH1
Variant allele fraction
0.0 0.1 0.2 0.3 0.4 0.5 0.6 0.7
Dx
Rel
N
AML_062
SMC1A DNMT3A NPM1 FLT3(p.D835Y) STAG2
KRAS KMT2A FLT3(p.S451F) IDH1
Variant allele fraction
0.0 0.2 0.4 0.6 0.8 1.0
Dx
Rel
O
AML_065
IDH2 DNMT3A NRAS NPM1
Variant allele fraction
0.0 0.1 0.2 0.3 0.4 0.5 0.6 0.7
Dx
Rel
P
AML_067
U2AF1 PTPN11 ASXL1 RUNX1
BRAF IDH2 TP53
Variant allele fraction
0.0 0.1 0.2 0.3 0.4 0.5 0.6 0.7
Dx
Rel
Q
AML_068
DNMT3A IDH2 NPM1 NRAS FLT3-ITD
Variant allele fraction
0.0 0.2 0.4 0.6 0.8 1.0
Dx
Rel
R
AML_069
WT1 EP300 FLT3-ITD
Variant allele fraction
0.0 0.2 0.4 0.6 0.8 1.0
Dx
Rel

S

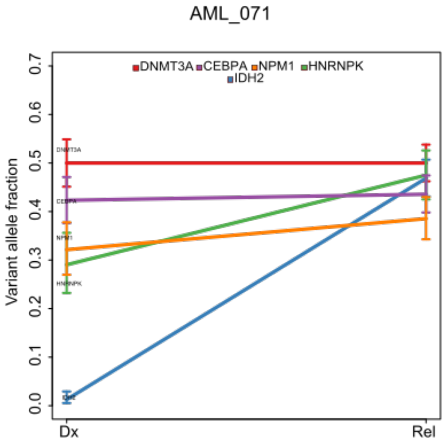

Supplementary Figure 6: Line plots of mutational composition for study subjects with clonal evolution patterns. VAFs at diagnosis and relapse are plotted for each mutation. Bars denote 95% confidence intervals for the VAF, accounting for the sequencing depth at the associated genomic positions, assuming a binomial distribution of the reads.

Supplementary Figure 7

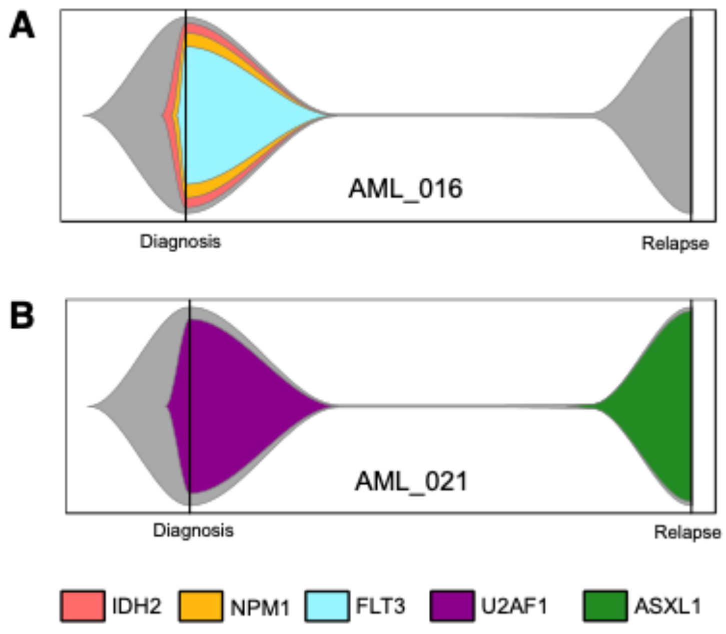

Supplementary Figure 7: Graphical representations of clonal evolution patterns. A and **B.** Fish plots of representative patients which undergo clonal evolution between diagnosis and relapse are shown. The color key for gene contributions to the pattern is located in the lower right corner of the figure. Each vertical line indicates a tumor sample at diagnosis or relapse.

Supplementary Figure 8

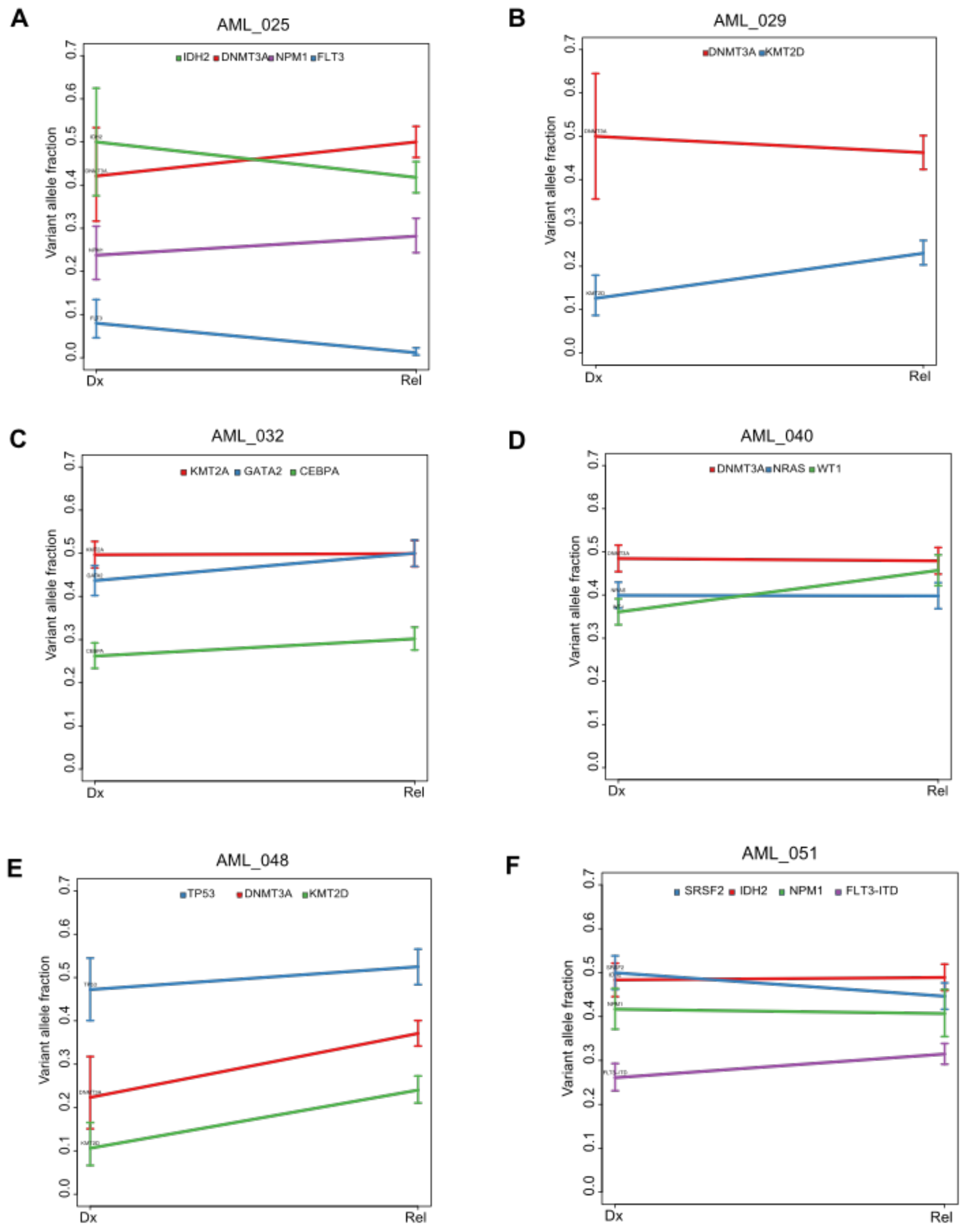

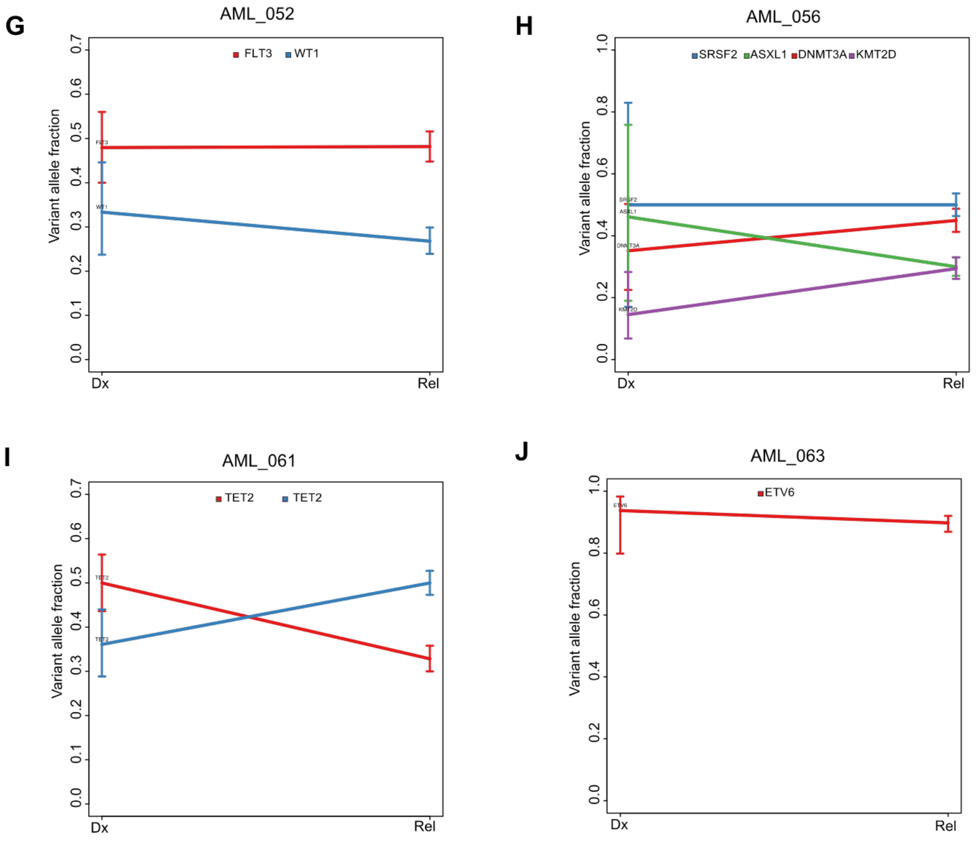

Supplementary Figure 8: Line plots of mutational composition for study subjects with stable evolution patterns.

VAFs at diagnosis and relapse are plotted for each mutation. Bars denote 95% confidence intervals for the VAF, accounting for the sequencing depth at the associated genomic positions, assuming a binomial distribution of the reads.

Supplementary Figure 9

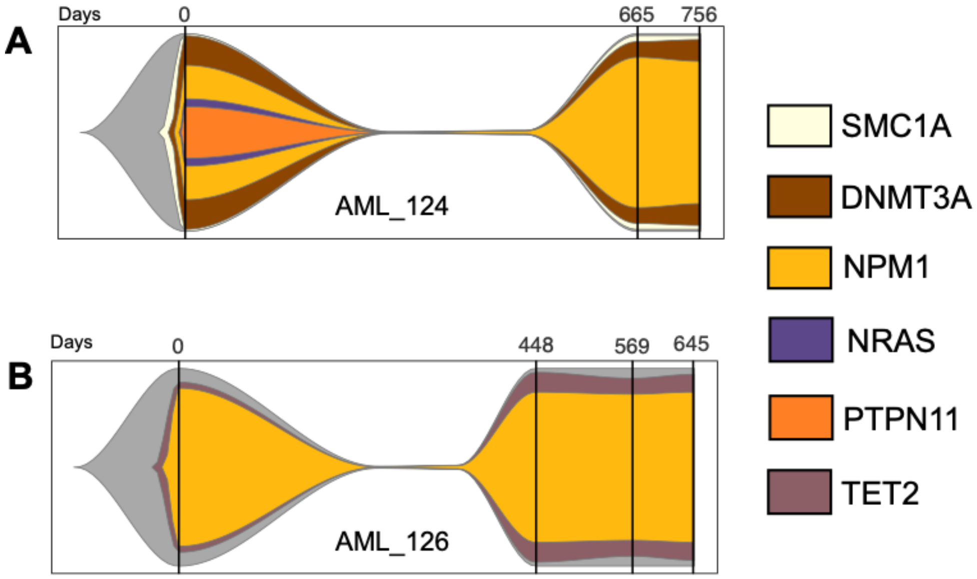

Supplementary Figure 9: Graphical representations of clonal evolution patterns in serial specimens. A and **B.** Fish plots representing tumor evolution patterns in patients from whom serial specimens were available are shown. The color key for gene contributions to the pattern is located in the lower right corner of the figure. Each vertical line indicates a tumor sample, and the x-axis represents time (days) from the diagnosis stage of disease.

Supplementary Tables

Supplementary Table 1: Summary of study subject clinical data.
Table of clinical characteristics of patients in the study cohort. Included parameters are: Study subject identification numbers (Subject ID) match the assigned identifiers in dbGap accession number phs001027.v2.p1 (Subject_ID assignments). M = male, F = female; Age at diagnosis (years); clinical results of cytogenetic assessments and recurrent cytogenetic events (Cytogenetics at diagnosis) at the diagnosis stage; ELN = European Leukemia Net risk stratification(1); Induction and consolidation treatments each study subject received after diagnosis (Ara-C = cytarabine arabinoside; AlloSCT = Allogeneic stem cell transplant; AutoSCT = Autologous stem cell transplant); Time Until relapse (days between disease diagnosis and relapse); Genomic assays performed on DNA from each study subject (1 = performed; blank = not performed); Reference IDs (specimen IDs for diagnosis specimens subjected to targeted resequencing [EGAS00001000275]) from (11).

Supplementary Table 2: Somatic mutations determined in study subjects.
Summary table of all somatic mutations detected in study cohort. Somatic mutation results are from both Exome Capture (Exome; sheet ST2a Exome) and Targeted panel resequencing (sheet ST2b Targeted panel sequencing results from the entire cohort, and sheet ST2c Targeted panel sequencing results from serial AML specimens) assays. Included are the expected amino acid changes and VAF.

Supplementary Table 3: Mutations identified in the whole exome sequencing cohort and associated changes during AML disease progression.
Each line is a single somatic mutation identified in a single study subject. The columns are: study subject identification numbers, the gene name, the amino acid annotation, the chromosome, the genomic location, the reference allele, the alternative allele, the VAF at diagnosis, the VAF at relapse and if this mutation was gained (GA), lost (LO) or stable (ST) at relapse compared to diagnosis.

Supplementary Table 4: Somatic mutations validated in the whole exome sequencing cohort.
Each line is a single mutation identified in a single study subject. The columns are: study subject identifier, if the sample was the diagnosis or the relapse sample, the chromosome name, the genomic location, the reference allele, the alternative allele, the gene name, the HGSVc effect of the mutation, the HGSVp effect of the mutation, the exon of the gene the specified mutation is located in, the allele frequency in ExAC, the DBSNP GMAF allele frequency, the number of times this mutation has been reported in COSMIC, the TCGA mutation ID, the number of reads that contain the alternative allele, the sequencing depth at this position and the variant allele fraction.

Specimen ID	IDH1		IDH2	
	Diagnosis	Relapse	Diagnosis	Relapse
AML_102	IDH1-WT	IDH1-WT	IDH2-WT	IDH2-WT
AML_103	IDH1-WT	IDH1-WT	IDH2-WT	IDH2-WT
AML_104	IDH1-WT	IDH1-WT	IDH2-R172K	IDH2-R172K
AML_105	IDH1-WT	IDH1-WT	IDH2-R140Q	IDH2-R140Q
AML_108	IDH1-WT	IDH1-WT	IDH2-WT	IDH2-R140Q
AML_109	IDH1-WT	IDH1-WT	IDH2-WT	IDH2-WT
AML_113	IDH1-WT	IDH1-WT	IDH2-R140Q	IDH2-R140Q
AML_114	IDH1-WT	IDH1-WT	IDH2-R140Q	IDH2-R140Q
AML_116	IDH1-WT	IDH1-WT	IDH2-R140Q	IDH2-R140Q
AML_118	IDH1-WT	IDH1-WT	IDH2-WT	IDH2-WT
AML_119	IDH1-WT	IDH1-WT	IDH2-R140Q	IDH2-R140Q
AML_121	IDH1-WT	IDH1-WT	IDH2-WT	IDH2-WT
AML_124	IDH1-WT	IDH1-WT	IDH2-WT	IDH2-WT
AML_126	IDH1-WT	IDH1-WT	IDH2-WT	IDH2-WT
AML_127	IDH1-WT	IDH1-WT	IDH2-WT	IDH2-WT
AML_128	IDH1-R132H	IDH1-R132H	IDH2-WT	IDH2-WT
AML_130	IDH1-WT	IDH1-WT	IDH2-WT	IDH2-WT
AML_138	IDH1-WT	IDH1-WT	IDH2-WT	IDH2-WT
AML_139	IDH1-WT	IDH1-WT	IDH2-WT	IDH2-WT

Supplementary Table 5: IDH1 and IDH2 mutation validations using a sequenom assay. A subset of specimens were processed using a sequenom assay to detect IDH1-R132H, IDH2-R172K and IDH2-R140Q mutations. WT = wild type.

Supplementary Table 6: Copy number aberration analysis results.
Each line is a CNA or CNA pattern identified in a single study subject. Normal = normal cytogenetics; Disease stage = determination of if CNA event was detected at Diagnosis only (diagnosis), Relapse only (relapse) or at both stages of the disease (both).amp = amplification; del = deletion

Supplementary Table 7: Mutations identified in the targeted panel sequencing cohort.
Each line is a single mutation identified in a single study subject. The columns are: study subject identification numbers, the gene name, the amino acid annotation, the mutation significance (YES = oncogenic; ONCOGENIC, LIKELY = likely oncogenic, or UNKNOWN = unknown significance), the chromosome, the genomic location, the reference allele, the alternative allele, the VAF at diagnosis, the depth of coverage at diagnosis, the clone number is the clone that the mutation belongs to in the diagnosis sample (0 denoting a clone that is not present in the diagnosis sample), the VAF at relapse, the depth of coverage at relapse and the clone number is the clone that the mutation belongs to in the relapse sample (0 denoting a clone that is not present in the relapse sample). Gray coloring of the VAF and the depth of a mutation at diagnosis (respectively at relapse) indicates specimens in which the VAF and depth were corrected for low tumor content in the diagnosis (respectively relapse) sample.

Supplementary Table 8: Inferred clonal evolution groups.
Subject ID = study subject identification number. EvolutionModel.group = results of inferred evolution model analysis. EpialleleCluster = annotated epigenetic evolution progression for each subject identified in (2).

Supplementary Table 9: Multi-relapse patient specimen information and results.
Targeted panel sequencing was performed on serial specimens from three patients. Sheet ST9a: Shown are the collection days relative to the date on which diagnosis specimens were collected. NA = not applicable. Sheet ST9b: mutations identified in the targeted panel sequencing performed on the multi-relapse specimens. Each line is a single mutation identified in a single study subject. The columns are: study subject identification numbers, the gene name, the amino acid annotation, the mutation significance (YES = oncogenic; ONCOGENIC, LIKELY = likely oncogenic, or UNKNOWN = unknown significance), predicted mutation effect, the chromosome, the genomic location, the reference allele, the alternative allele, the VAF detected, the depth of coverage, and the clone number indicates the clone that the mutation belongs to at first occurrence.

Group	pvalue
Age_at_Dx	0.471725282032799
Sex	0.753196705256616
EpialleleCluster	0.433359563026832
ELN	0.153662863192993
Treatment	0.736459472262838
Time_to_relapse	0.280350482159572

Supplementary Table 10: Kruskal Wallis tests results assessing for association between genomic evolution categories, DNA methylation epiallele categories, and clinical parameters.
Group = clinical parameter assessed . p-value = *P*-value from the Kruskal wallis test.

Supplementary Table 11: Sequencing statistics of the sparse whole genome sequencing assay performed in the study.

Specimen ID = study subject identification number; Disease stage indicates if the sequencing results are from the diagnosis or relapse stage; TOTAL = total number of reads; Number of duplicate reads = number of reads removed from analysis due to them being assessed to be duplicate reads; Percent mapped reads = percent of total reads that mapped uniquely to the genome and utilized in analysis; Mean coverage per base in mapped genomic regions = mean coverage per base after alignment of the reads to reference genome.

Supplementary Materials' bibliography

1. Dohner H, Estey E, Grimwade D, Amadori S, Appelbaum FR, Buchner T, et al. Diagnosis and management of AML in adults: 2017 ELN recommendations from an international expert panel. Blood. 2017;129(4):424-47.
2. Li S, Garrett-Bakelman FE, Chung SS, Sanders MA, Hricik T, Rapaport F, et al. Distinct evolution and dynamics of epigenetic and genetic heterogeneity in acute myeloid leukemia. Nat Med. 2016;22(7):792-9.
3. Li H, Durbin R. Fast and accurate long-read alignment with Burrows-Wheeler transform. Bioinformatics. 2010;26(5):589-95.
4. Cibulskis K, Lawrence MS, Carter SL, Sivachenko A, Jaffe D, Sougnez C, et al. Sensitive detection of somatic point mutations in impure and heterogeneous cancer samples. Nat Biotechnol. 2013;31(3):213-9.
5. Koboldt DC, Zhang Q, Larson DE, Shen D, McLellan MD, Lin L, et al. VarScan 2: somatic mutation and copy number alteration discovery in cancer by exome sequencing. Genome Res. 2012;22(3):568-76.
6. Larson DE, Harris CC, Chen K, Koboldt DC, Abbott TE, Dooling DJ, et al. SomaticSniper: identification of somatic point mutations in whole genome sequencing data. Bioinformatics. 2012;28(3):311-7.
7. Ye K, Schulz MH, Long Q, Apweiler R, Ning Z. Pindel: a pattern growth approach to detect break points of large deletions and medium sized insertions from paired-end short reads. Bioinformatics. 2009;25(21):2865-71.
8. McKenna A, Hanna M, Banks E, Sivachenko A, Cibulskis K, Kernytsky A, et al. The Genome Analysis Toolkit: a MapReduce framework for analyzing next-generation DNA sequencing data. Genome Res. 2010;20(9):1297-303.
9. Cingolani P, Platts A, Wang le L, Coon M, Nguyen T, Wang L, et al. A program for annotating and predicting the effects of single nucleotide polymorphisms, SnpEff: SNPs in the genome of Drosophila melanogaster strain w1118; iso-2; iso-3. Fly (Austin). 2012;6(2):80-92.
10. Kim SY, Speed TP. Comparing somatic mutation-callers: beyond Venn diagrams. BMC Bioinformatics. 2013;14:189.
11. Papaemmanuil E, Gerstung M, Bullinger L, Gaidzik VI, Paschka P, Roberts ND, et al. Genomic Classification and Prognosis in Acute Myeloid Leukemia. N Engl J Med. 2016;374(23):2209-21.
12. Lek M, Karczewski KJ, Minikel EV, Samocha KE, Banks E, Fennell T, et al. Analysis of protein-coding genetic variation in 60,706 humans. Nature. 2016;536(7616):285-91.
13. Desai P, Mencia-Trinchant N, Savenkov O, Simon MS, Cheang G, Lee S, et al. Somatic mutations precede acute myeloid leukemia years before diagnosis. Nat Med. 2018;24(7):1015-23.
14. Perugini M, Iarossi DG, Kok CH, Cummings N, Diakiw SM, Brown AL, et al. GADD45A methylation predicts poor overall survival in acute myeloid leukemia and is associated with IDH1/2 and DNMT3A mutations. Leukemia. 2013;27(7):1588-92.
15. Baslan T, Kendall J, Rodgers L, Cox H, Riggs M, Stepansky A, et al. Genome-wide copy number analysis of single cells. Nat Protoc. 2012;7(6):1024-41.
16. Baslan T, Kendall J, Ward B, Cox H, Leotta A, Rodgers L, et al. Optimizing sparse sequencing of single cells for highly multiplex copy number profiling. Genome Res. 2015;25(5):714-24.
17. FASTQC 2012 [Available from: https:// www.bioinformatics.babraham.ac.uk/projects/fastqc/).
18. Martin M. Cutadapt removes adapter sequences from high-throughput sequencing reads. 2011. 2011;17(1):3.

19. Li H, Handsaker B, Wysoker A, Fennell T, Ruan J, Homer N, et al. The Sequence Alignment/Map format and SAMtools. Bioinformatics. 2009;25(16):2078-9.
20. Picard Tools [Available from: https://broadinstitute.github.io/picard/.
21. BedTools [Available from: https://bedtools.readthedocs.io/en/latest/.
22. Olshen AB, Venkatraman ES, Lucito R, Wigler M. Circular binary segmentation for the analysis of array-based DNA copy number data. Biostatistics. 2004;5(4):557-72.
23. Seshan VE, Olshen AB. DNAcopy: A Package for Analyzing DNA Copy Data 2019 [Available from: https://bioconductor.org/packages/release/bioc/html/DNAcopy.html.
24. Benaglia T, Chauveau D, Hunter DR, Young DS. mixtools: An R Package for Analyzing Mixture Models. 2009. 2009;32(6):29.
