# Supplementary material for: Genomic and evolutionary portraits of disease relapse in acute myeloid leukemia
Source: Leukemia. 2021 Feb 12;35(9):2688–92. doi: 10.1038/s41375-021-01153-0 (PMC8357838; doi:10.1038/s41375-021-01153-0)
Supplement: Supplementary file 8 — Supplementary Table 8 [file 41375_2021_1153_MOESM8_ESM.docx]

| **Supplementary Table 8. Inferred clonal evolution groups.** | | |
| --- | --- | --- |
|  |  |  |
| **Subject ID** | **EvolutionModel.group** | **EpialleleCluster** |
| AML_003 | Clonal changes | 3 |
| AML_004 | Clonal changes | 1 |
| AML_011 | Clonal changes | 1 |
| AML_012 | Clonal changes | 2 |
| AML_016 | Clonal changes | 1 |
| AML_018 | Clonal changes | 1 |
| AML_019 | Clonal changes | 2 |
| AML_021 | Clonal changes | 1 |
| AML_023 | Clonal changes | 2 |
| AML_024 | Clonal changes | 3 |
| AML_034 | Clonal changes | 3 |
| AML_036 | Clonal changes | 2 |
| AML_037 | Clonal changes | 1 |
| AML_062 | Clonal changes | 1 |
| AML_065 | Clonal changes | 3 |
| AML_067 | Clonal changes | 1 |
| AML_068 | Clonal changes | 3 |
| AML_069 | Clonal changes | 3 |
| AML_071 | Clonal changes | 2 |
| AML_025 | Stable | 1 |
| AML_029 | Stable | 3 |
| AML_032 | Stable | 1 |
| AML_040 | Stable | 1 |
| AML_048 | Stable | 2 |
| AML_051 | Stable | 3 |
| AML_052 | Stable | 1 |
| AML_056 | Stable | 2 |
| AML_061 | Stable | 1 |
| AML_063 | Stable | 2 |
| AML_001 | Subclonal changes | 1 |
| AML_002 | Subclonal changes | 1 |
| AML_005 | Subclonal changes | 1 |
| AML_006 | Subclonal changes | 3 |
| AML_007 | Subclonal changes | 1 |
| AML_008 | Subclonal changes | 1 |
| AML_010 | Subclonal changes | 3 |
| AML_014 | Subclonal changes | 1 |
| AML_015 | Subclonal changes | 3 |
| AML_020 | Subclonal changes | 1 |
| AML_026 | Subclonal changes | 3 |
| AML_027 | Subclonal changes | 3 |
| AML_028 | Subclonal changes | 3 |
| AML_031 | Subclonal changes | 1 |
| AML_033 | Subclonal changes | 2 |
| AML_035 | Subclonal changes | 2 |
| AML_042 | Subclonal changes | 1 |
| AML_043 | Subclonal changes | 1 |
| AML_046 | Subclonal changes | 1 |
| AML_049 | Subclonal changes | 3 |
| AML_050 | Subclonal changes | 1 |
| AML_053 | Subclonal changes | 1 |
| AML_055 | Subclonal changes | 2 |
| AML_057 | Subclonal changes | 1 |
| AML_058 | Subclonal changes | 2 |
| AML_059 | Subclonal changes | 3 |
| AML_060 | Subclonal changes | 2 |
| AML_064 | Subclonal changes | 1 |
| AML_070 | Subclonal changes | 1 |
| AML_072 | Subclonal changes | 2 |
| AML_073 | Subclonal changes | 2 |
| AML_022 | Unknown | 1 |
| AML_038 | Unknown | 1 |
| AML_041 | Unknown | 1 |
